# MOPA: An integrative multi-omics pathway analysis method for measuring omics activity

**DOI:** 10.1371/journal.pone.0278272

**Published:** 2023-03-16

**Authors:** Jaemin Jeon, Eon Yong Han, Inuk Jung

**Affiliations:** 1 School of Computer Science and Engineering, Kyungpook National University, Buk-gu, Deagu, Republic of Korea; 2 Interdisciplinary Program in Bioinformatics, Seoul National University, Gwanak-Gu, Seoul, Republic of Korea; Chinese Academy of Sciences, CHINA

## Abstract

Pathways are composed of proteins forming a network to represent specific biological mechanisms and are often used to measure enrichment scores based on a list of genes in means to measure their biological activity. The pathway analysis is a de facto standard downstream analysis procedure in most genomic and transcriptomic studies. Here, we present MOPA (Multi-Omics Pathway Analysis), which is a multi-omics integrative method that scores individual pathways in a sample wise manner in terms of enriched multi-omics regulatory activity, which we refer to mES (multi-omics Enrichment Score). The mES score reflects the strength of regulatory relations between multi-omics in units of pathways. In addition, MOPA is able to measure how much each omics contribute to mES that may be used to observe what kind of omics are active in a pathway within a sample group (e.g., subtype, gender), which we refer to OCR (Omics Contribution Rate). Using nine different cancer types, 93 clinical features and three types of omics (i.e., gene expression, miRNA and methylation), MOPA was used to search for clinical features that were explainable in context of multi-omics. By evaluating the performance of MOPA, we showed that it yielded higher or at least equal performance compared to previous single and multi-omics pathway analysis tools. We find that the advantage of MOPA is the ability to explain pathways in terms of omics relation using mES and OCR. As one of the results, the TGF-beta signaling pathway was captured as an important pathway that showed distinct mES and OCR values specific to the CMS4 subtype in colon adenocarcinoma. The mES and OCR metrics suggested that the mRNA and miRNA expressions were significantly different from the other subtypes, which was concordant with previous studies. The MOPA software is available at https://github.com/jaeminjj/MOPA.

## Introduction

With the growing interest in finding meaningful correlations across different omics data types, the size of multi-omics data is growing exponentially. Such multi-omics data are sampled and analyzed in means to explain certain clinical outcomes or biological phenomena of interest.

Advancement in methods for measuring such omics data is outpacing the development of analytical methods that are able to embrace the many omics types in an integrative manner. The multi-omics research has now become a routine that is rapidly extending into various domains, such as, metagenomics and single-cell [1]. While the advantage of utilizing multi-omics data is evident, the complexity of the analysis is non-trivial. Especially, as multi-omics, or multi-modal, data provide a multi-perspective view to a common event, the interpretation of the result can become relatively difficult. There are a number of valuable resources providing large scale multi-omics data, such as TCGA [2], ENCODE [3] and GTEx [4].

Recently, a number of methods has been proposed that perform integrative analysis using multi-omics data. For example SNF [5], MOFA [6] and iCluster+ [7] are able to integrate a wide range of omics data types as is. MONTI [8], CNAme [9], iGC [10] and MethylMix [11] are also multi-omics analysis methods, but perform the analysis on gene-level transformed data. These methods commonly require the omics data to be sample matched. In many cases, the output is also in units of genes or omics features. To make biological interpretations, a list of omics features need to be further investigated, which frequently involves enrichment tests, such as GSVA [12] or GSEA [13].

Instead of a list of omics features, it would be more helpful if the output was in units of pathways, since a pathway itself represent some biological mechanism, and thus is self explainable. Currently, three multi-omics methods were found whose output is in units of pathways, where one was able to compute pathway scores for each sample. The three methods are ActivePathway [14], multiGSEA [15] and MOGSA (Multi omics pathway Geneset Analysis) [16]. ActivePathway and multiGSEA methods take multi-omics data as input and output p-values for each pathway. ActivePathway computes the p-value of a pathway using a ranked hypergeometric test for each omics, which are later combined as a single p-value. multiGSEA is an extension of GSEA, where it performs the conventional enrichment analysis on each omics data. Similar to ActivePathway, multiGSEA combines the pathway p-values of each omics into a single p-value. On the other hand, MOGSA produces pathway enrichment scores per sample by performing multivariate analysis for dimension reduction to detect latent multi-omics features. In comparison to the multi-omics approaches, GSVA, ssGSEA [17] and z-score [18] are frequently used methods that output pathway enrichment scores based on the single-omics gene expression data. The characteristics of a number of pathway analysis methods are compared in Table 1.

**Table 1:** Comparison of multi-omics pathway analysis methods.

Method	Supporting omics	Analysis target	Output
MOPA	multi-omics	Single sample	Scoring matrix
MOGSA	multi-omics	Single sample	Scoring matrix
ActivePathways	multi-omics	Group	p-value
multiGSEA	multi-omics	Group	p-value
GSVA	single-omics	Single sample	Scoring matrix
GSEA	single-omics	Group	Scoring matrix
ssGSEA	single-omics	Single sample	Scoring matrix
z-score	single-omics	Single sample	Scoring matrix

Moreover, since pathway results are used to understand the biological mechanism of a disease or phenotype of interest, pathway scores or p-values are expected to associate with underlying biological semantics of a given experimental condition or clinically available features, such as cancer subtype, gender or age. However, the majority of tools are based on unsupervised methods that aim to discover new groups that exhibit group specific multi-omics profiles.

Here, we propose MOPA (Multi-Omics Pathway Analysis), which is a tool that takes multi-omics data as input and outputs a score of a pathway per sample in association to a phenotype or clinical feature. A high scored pathway implies that the multi-omics profiles of genes within the pathway are different between the clinical feature groups of interest, which therefore may explain their biological difference in units of pathway and in context of multi-omics relation. The performance of MOPA was evaluated using multi-omics data from nine cancer types and 93 clinical features, followed by two detailed use case studies on colon and stomach adenocarcinoma cohorts. Compared to pathway based analysis tools, MOPA showed equal or improved classification performance. Distinct from other methods, MOPA is able to rank pathways by their association strength to a given target label. Most importantly, it provides mES (multi-omics Enrichment Score) and OCR (Omics Contribution Rate) metrics as output that represent the enrichment of multi-omics activity and the contribution of each omics towards the activity in each pathway. The mES and OCR metrics were developed to be used in means to enhance the comprehension of the pathway analysis result.

The mES score is a single value that integrates all three omics. The mES score is computed for each pathway in a single sample. A high mES score indicates that a certain regulatory relationship between the three omics is significantly enriched in a pathway. For example, if genes are strongly suppressed by miRNAs within a pathway, a regulatory relation between mRNA and miRNA omics is present and the pathway will have a high mES. If this was true for the omics relation between mRNA and methylation, a high mES will be computed. Hence, mES indicates the strength of any combination of omics. To supplement the mES, we defined the second metric OCR, which is able to show specifically what type of omics relations are taking part in the mES score.

Using mES and OCR, we illustrated that MOPA was able to detect pathways that were concordant with previously reported results in colon and stomach adenocarcinoma studies in context of multi-omics.

## Materials and methods

### Ethics statement

The multi-omics data of patients from various cohorts in the TCGA data portal was used. The type of cohorts, number of patients and the clinical features used in this study are provided in the S1 Table. All the data are level 3 data that are publicly available. The controlled access level 1 data was not used. Every patient is de-identified and encoded with TCGA sample codes. The related clinical information of patients also do not contain any private information. The results shown in this study are in whole or part based upon data generated by the TCGA Research Network: https://www.cancer.gov/tcga. All the data satisfy the freedom-to-public criteria stated at https://www.cancer.gov/about-nci/organization/ccg/research/structural-genomics/tcga/using-tcga/citing-tcga.

### Multi-omics data set

**Fig 1:**
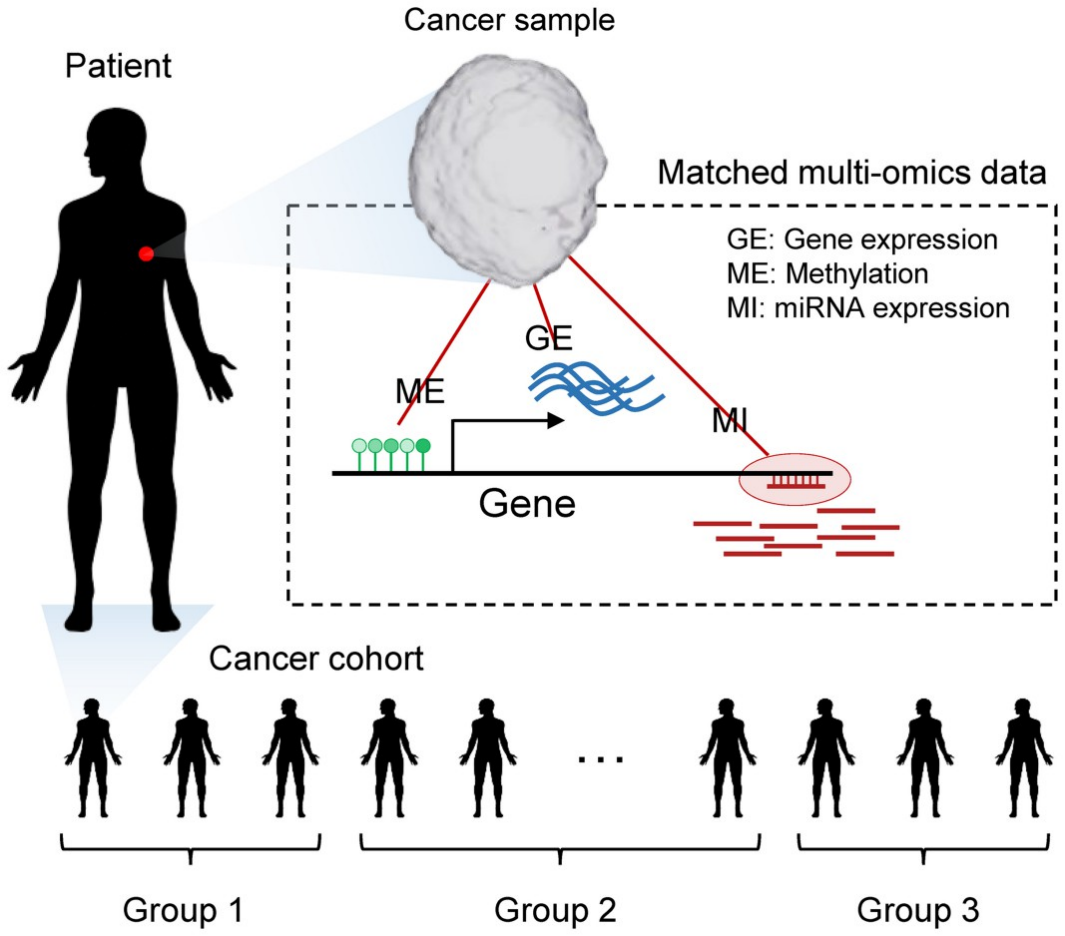
Description of patient matched multi-omics data. Multi-omics data are collected per patient in a multi-modal manner.

The multi-omics data was collected in a case, or patient, matched manner. Thus, cases with matching omics of interest were selected and processed as shown in Fig 1. Here, the groups refer to some clinical features within a cohort that serve as target labels to be predicted by the multi-omics enrichment scores (mES) of pathways computed by MOPA. The details of data preprocessing are described in our previous work [8]. In brief, the multi-omics data from the same set of patients are processed in means to capture gene-regulatory relations between the different omics that significantly differ between the groups.

Each omics layer is composed of different number of features, scale and data type, where the unit of features correspond to the omics specific elements, such as genes, miRNAs or methylation probes. MOPA focuses on detecting gene-regulatory cis-relations across the multi-omics layers. Hence, it is advantageous to convert each omics data into gene-level data, which is also natural for pathway analysis where each node in a pathway network corresponds to a protein or gene. This will result in a transformed data where each omics layer are now in unit of genes and have the same dimensions (i.e., equal set of patients and genes). Due to the massive number of possible combinations of trans-regulatory elements and their targets, it is difficult to create an informative gene × sample × omics tensor, since only a small portion will be meaningful. Hence in this study, the MOPA tool focuses on cis-regulatory elements.

**Fig 2:**
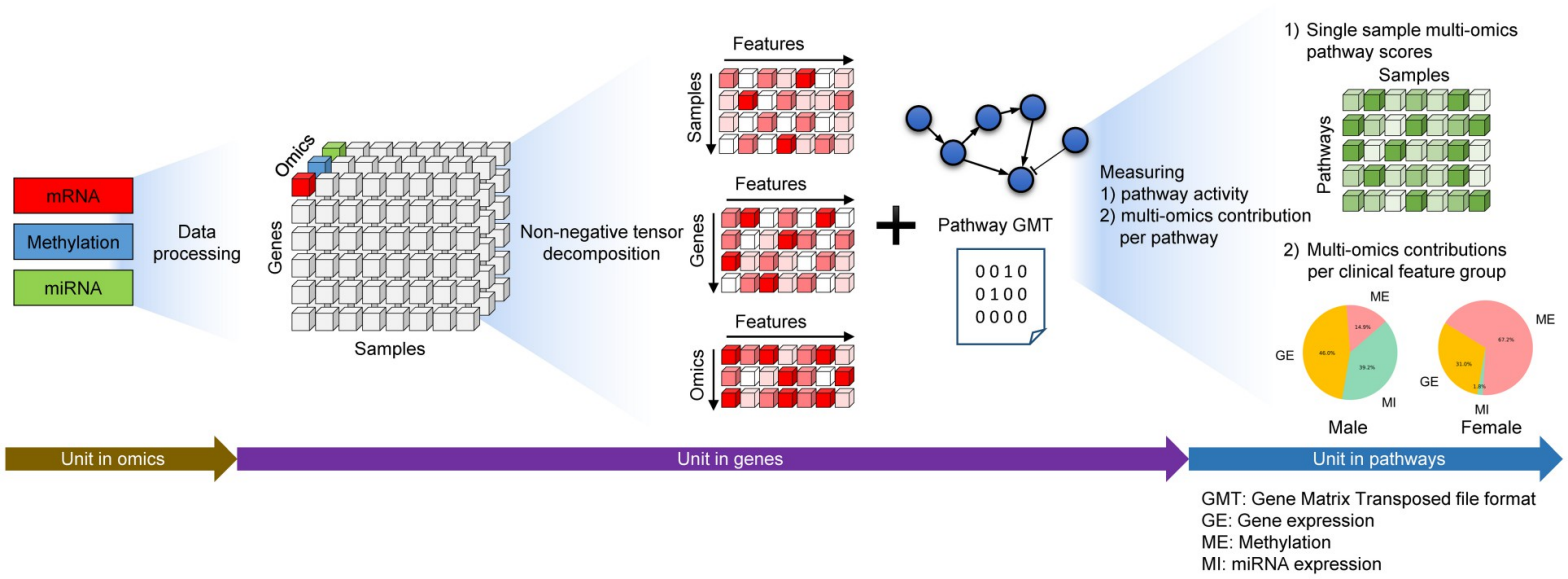
The conceptual workflow of MOPA. The sequential conversion of omics to gene and then to pathway level enrichment scores is shown.

Such property allows us to combine the omics slices into a tensor, or cube, which can be decomposed using a non-negative tensor decomposition method to find latent features. For searching latent features, we used MONTI (Multi-Omics Non-negative Tensor decomposition method for Integrative analysis) [8], which we have recently developed. MONTI takes a multi-omics tensor as input and outputs a subset of selected features that are highly associated to a clinical feature of interest. Based on the selected features, MOPA computes a pathway enrichment score per pathway and per sample, which is finally in units of pathways. In case where target labels are not available, MOPA does not utilize MONTI but searches for clusters with distinct multi-omics patterns. The workflow of MOPA starts by preprocessing omics data to convert them into gene-level data, from which informative features are selected to compute mES and OCR. It is a sequential process of converting the units of omics into gene-level data and finally into pathway-level enrichment scores as depicted in Fig 2.

MOPA was applied to nine cancer types using mRNA, miRNA and methylation data to predict the outcomes of 93 clinical features using mES. We also aimed to show that the interpretation of the result was advantageous over other methods using OCR. Thus, we selected methods that were able to score pathways in a sample wise manner since the scores reflect the omics activity in a pathway. Collectively, the performance of MOPA was evaluated by comparing the classification accuracy on clinical target outcomes to four pathway scoring methods, of which three are based on single-omics and one multi-omics data. While our interest lies within multi-omics, we included the single-omics pathway analysis tools to show the strength of multi-omics utilization. Among the multi-omics pathway tools, only MOGSA provided a scoring matrix as output. At last, case studies were performed on the colon and stomach adenocarcinoma cohorts data.

#### Cancer dataset

Gene expression, miRNA expression and methylation level omics data of nine cancer types were collected from the TCGA [19]. Cases with matching omics data were used. The number of collected samples per cancer type were BRCA (768), COAD (295), HNSC (495), KIRC (317), LUAD (451), PRAD (491), STAD (335), THCA (497) and UCEC (427). Among the many clinical features, 93 categorical features were used for the association study. Since the label of clinical features were partially available, the set of samples for each clinical feature varied. Thus, a set of samples was compiled in respect to available clinical feature labels. Table 2 lists a partial set of the cancer dataset we used, showing the clinical features for each cancer type and their categorical values. The categorical values are referred to as clinical feature groups throughout the study. The detail of the full dataset is provided in the S1 Table.

**Table 2:** A subset of the cancer dataset.

Cancer type	Clinical feature	Clinical feature groups	No. of samples
COAD	Molecular Subtype	CMS1, CMS2, CMS3, CMS4	234
STAD	Molecular Subtype	CIN, EBV, GS, MSI	305
BRCA	Subtype	LumA, LumB, HER2, Basal	595
HNSC	Gender	female, male	298
PRAD	Methylation cluster	1, 2, 3, 4	328
KIRC	Gender	female, male	252
LUAD	Methylation signature	low, intermediate, high	181
THCA	BRAF mutation group	0, 1	490
UCEC	Mrna expression cluster	1, 2, 3	221

#### Use case study dataset

For validation and presentation of the utility of MOPA, two use case studies were performed on the molecular subtypes in colon and stomach adenocarcinoma cohorts. For each study, we showed that MOPA was able reproduce important biological results specific to the cancer type and its clinical feature groups reported in previous studies. Here, the clinical feature groups refer to attributes from the medical health records, such as cancer subtype, age, gender and cancer stage.

#### Pathway and annotation data

The human KEGG [20] pathway database was used as the pathway source. Pathways are comprised of genes and MOPA performs the pathway analysis in a gene-centric manner. Hence, miRNA and methylation beta values were quantified per gene. For miRNA, the average expression of miRNAs that target a specific gene was assigned to that gene. For methylation, the average beta value of probes that are located within 2Kbp upstream of a gene’s transcription start site was assigned to that gene. For genes with no associated miRNAs or methylation probes were assigned with zero. Since pathways are composed of mainly protein coding genes, non-coding genes were excluded. The miRDB [21] was used to group miRNAs per target gene. The methylation probe annotation data from Illumina were used to group probes that are located within the 2kb upstream region of each gene’s transcription start site (TSS).

### Methods

**Fig 3:**
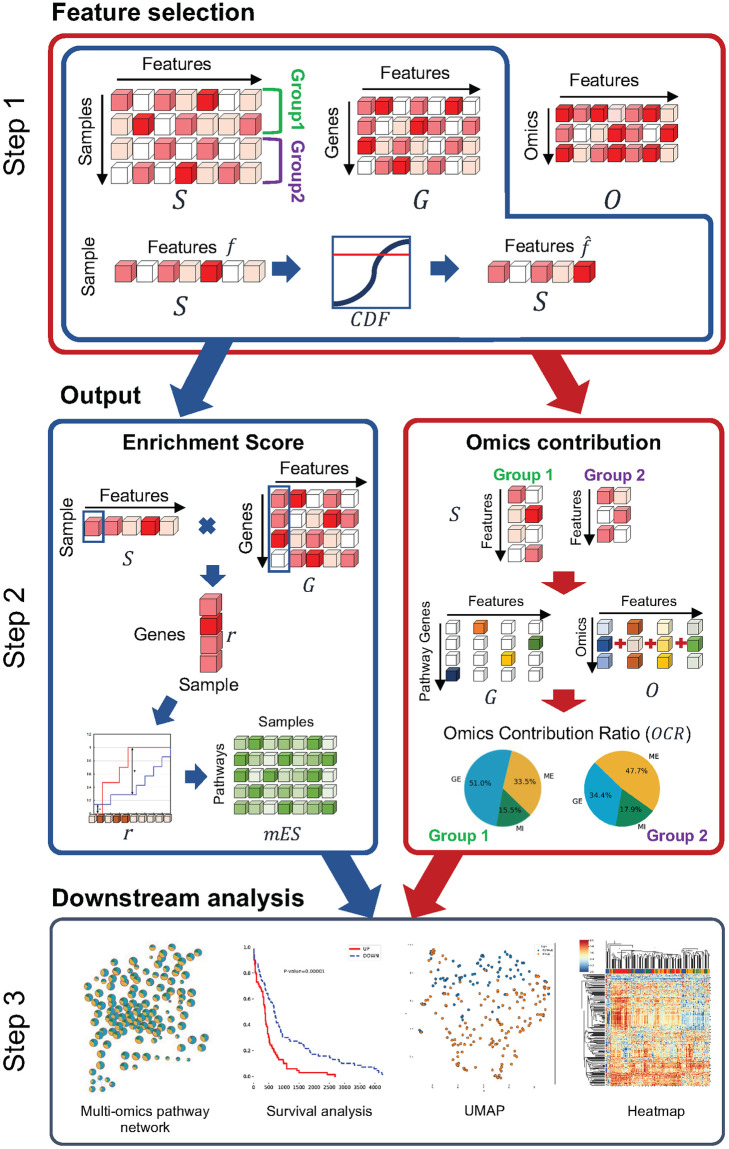
The three step workflow of MOPA for computing mES and OCR. The MOPA process is carried out in three steps. Step 1 performs feature selection to remove features with weak multi-omics information. Step 2 computes the mES and OCR based on the selected features in an independent manner. Step 3 performs biological interpretation that includes the construction of group specific pathway-to-pathway networks, survival analysis, UMAP and heatmap plotting.

The analysis workflow of MOPA consists of three steps as shown in Fig 3. In step 1, multi-omics data are preprocessed and analyzed to detect latent features in the gene-level. If clinical target labels are available, MOPA utilizes the result of MONTI. Otherwise, MOPA performs sample clustering on the mES score to create sample cluster labels. Here, the latent features represent distinct multi-omics patterns across the clinical feature groups or sample clusters in units of genes. In step 2, MOPA computes pathway enrichment scores based on the selected features, which also allows us to measure the contribution of each omics to the pathway enrichment score. In step 3, the resulting pathway enrichment scores are subject to downstream analysis, including ranking pathways and genes by their association strength to the sample labels, constructing multi-omics based pathway networks, survival analysis and visualization of the pathway scores.

#### Step 1: Sample wise gene-level multi-omics feature selection

Pathway scores are computed based on the latent features that are learned by the gene-level multi-omics integration method MONTI. MONTI takes a set of gene-level converted multi-omics data as input and outputs a list of latent gene features. In case when sample labels, or clinical feature labels, are available, MONTI will further select features that are highly associated to them. MONTI showed to be successful in selecting such features in various cancer types and clinical features. Otherwise, if labels are not available, MOPA skips the feature selection procedure in MONTI and proceeds the analysis in an unsupervised manner. The latent features are computed from the multi-omics tensor using the non-negative tensor factorization method PARAFAC [22], which maps the input multi-omics tensor data into a lower dimensional space. The tensor is decomposed into three feature components, each corresponding to an axis of the original input tensor (i.e., genes, samples and omics). The input to MOPA is a three-way array *T*, or a tensor, with genes *x*_*ijk*_, that is factorized into three loading matrices, *S*, *G*, and *O* with elements *s*_*if*_, *g*_*jf*_, and *o*_*kf*_. Here, matrices *S*, *G*, and *O* refer to the decomposed sample, gene, and omics components, respectively. Here, *i*, *j*, *k* and *f* refer to sample, gene, omics and rank feature indices, respectively. The tensor *T* is factorized using a predefined number of ranks *R*, which are referred to as the latent gene-level omics features. An optimal *R* for each dataset was predetermined based on the residual error of the decomposition and classification accuracy. If sample labels are available, MONTI selects informative features *f* among the *R* features, where *f* ⊂ *R*. Otherwise, all features are considered for further analysis, where *f* = *R*.

MOPA then assigns the features *f* to samples based on their values in the sample component *S*. A value *s*_*if*_ indicates the association, or weight, of feature *f* to sample *i*. Here, the task is to assign features to samples with high association and discard features with weak or no association to any sample. Thus, each sample may be assigned with a different set of features. However, samples within the same clinical feature group or sample cluster are expected to share a common set of features if their multi-omics profile share some common characteristics. To define a threshold for assigning features to samples, first, for each sample *i*, the vector *s*_*i*_ is min-max scaled to both normalize the feature values and prevent the case where a sample is not assigned with any feature by Eq 1. Min and Max function in Eq 1 helps to make minimum and maximum value in vector *s*_*i*_.

$$\begin{array}{c} s_{i}^{\prime }=\frac{s_{i}-min\left(s_{i}\right)}{max\left(s_{i}\right)-min\left(s_{i}\right)},\forall i\in Samples \end{array}$$ (1)

Then, the probability of features from the cumulative distribution function (CDF) was computed based on the scaled vector $s_{i}^{\prime }$ by Eq 2. *P*_*kde*_ function means kernel density estimation.

$$\begin{array}{c} CDF_{i}=\int_{-\infty }^{s_{i}^{\prime }}P_{kde}\left(s_{i}^{\prime }\right)dx \end{array}$$ (2)

**Fig 4:**
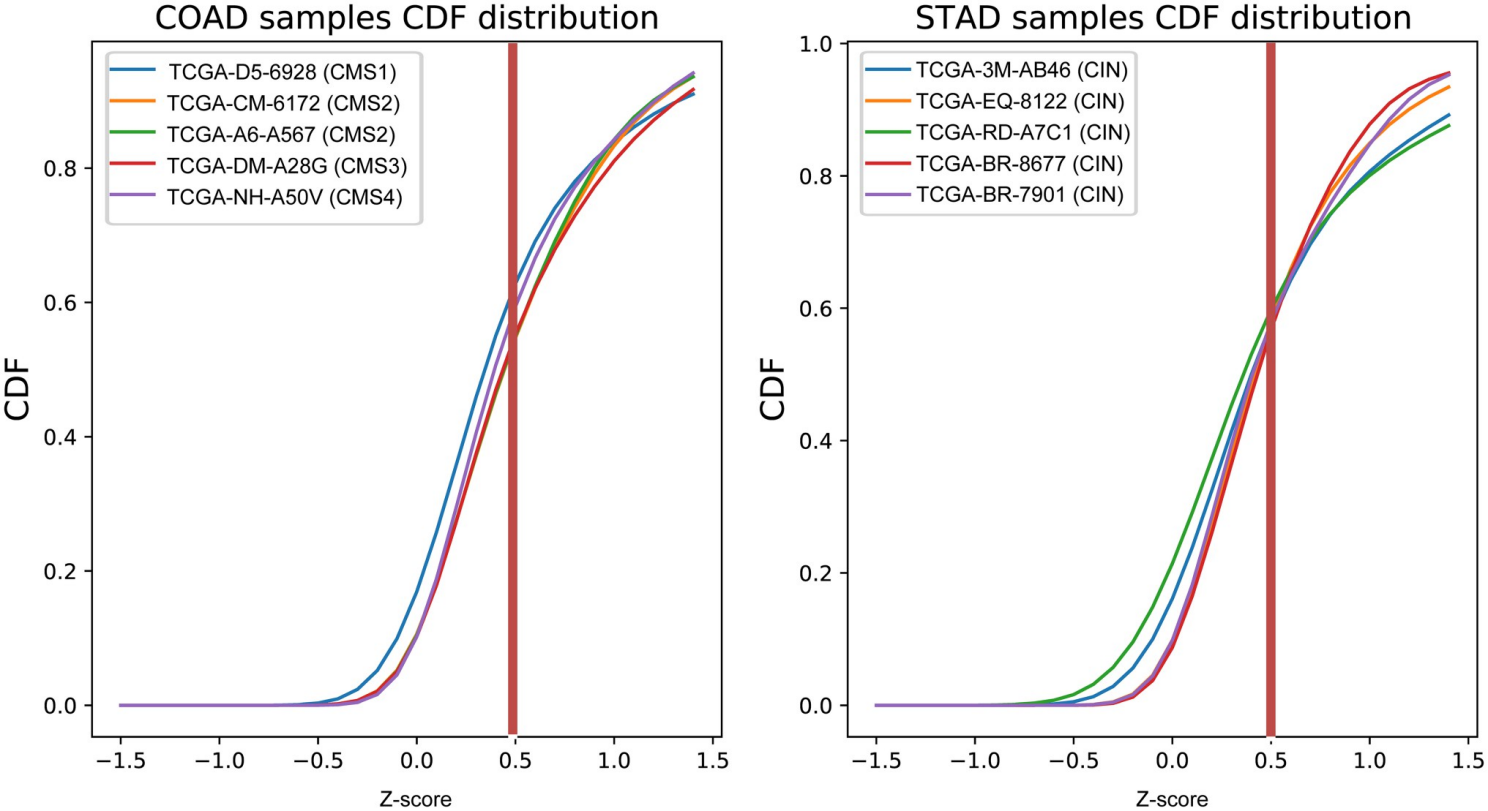
The CDF plots of a set of randomly selected STAD and COAD samples. Five samples were randomly selected for which the CDF plots were visualized. The horizontal line at 0.6 indicates the CDF threshold, that is used as the criteria for feature selection.

Now, rank features in sample *i* have cumulative probabilities, which is denoted as *CDF*_*i*_. The probability of a feature in *CDF*_*i*_ reflects its association strength to sample *i*. By testing a range of CDF thresholds for selecting informative features, 0.6 showed robust results across several different datasets as shown in Fig 4. Thus, 0.6 was used as the threshold for assigning a set of features $\hat{f}$ to sample *i*, where $\hat{f}\subseteq f$. The result will be a list of samples with their assigned rank features $s_{i\hat{f}}^{\prime }$, as shown in Fig 3 (Step 1). The final output of Step 1 is $s_{i\hat{f}}^{\prime }$, which is used to compute the sample wise pathway mES and OCR metrics in Step 2.

#### Step 2: Computing multi-omics pathway enrichment score and omics contribution rate

Here, the multi-omics Enrichment Score (mES) and Omics Contribution Rate (OCR) are computed. mES reflects the multi-omics signal strength of a pathway in each sample, whereas OCR indicates how much each omics contribute to mES.

First, to compute mES, three types of input data are required: 1) the decomposed sample and gene components, *S* and *G*, 2) a Gene Matrix Transposed (GMT) file in binary format indicating the gene memberships to pathways and 3) the sample assigned features $\hat{f}$. The mES is similar to the enrichment score in GSVA, where a high score indicates that a significantly large portion of genes with membership to a pathway of interest are highly activated in terms of multi-omics, compared to non-member genes. Conventionally, the enrichment score is computed based on a list of expression level ranked genes. To extend it to embed multi-omics data, a mixture of multi-omics features in $s_{i\hat{f}}^{\prime }$ and $g_{\hat{f}}^{\prime }$ are used in a single sample *i*. Here, $g_{\hat{f}}^{\prime }$, is the z-normalized and positive scaled result of $g_{\hat{f}}$. For each gene *j*, the sample feature values $s_{i\hat{f}}$ is multiplied by $g_{j\hat{f}}$, which are summed for all features $\hat{f}$ as in Eq 3.

$$\begin{array}{c} r_{ij}=\sum_{f=1}^{\hat{f}}g_{jf}^{\prime }\times s_{if}^{\prime } \end{array}$$ (3)

The above is computed for each gene and each sample, which is the matrix vector product ${\vec{r}}_{i}$ (Eq 4).

$$\begin{array}{c} {\vec{r}}_{i}={\left(g_{\hat{f}}^{\prime }\right)}^{T}\cdot s_{i\hat{f}}^{\prime } \end{array}$$ (4)

Here, ${\vec{r}}_{i}$ is sorted to order genes, which is the input for computing mES using the Kolmogorov–Smirnov (KS) random walk statistic. While multiple omics are used, genes are ordered by a single value ${\vec{r}}_{ij}$. This is because ${\vec{r}}_{ij}$ embeds the sample specific multi-omics characteristics $s_{if}^{\prime }$ as shown in Eq 3. Similar to GSVA, the objective is to measure the similarity of two distributions. Here, the two distributions are one that consists of genes with membership to a pathway, and another with genes without membership. The result will be the cumulative difference, *d*_*ijt*_, up to the *j*-th ordered gene between the two distributions of pathway *t* in sample *i* as shown in Eq 5,

$$\begin{array}{c} d_{ijt}=\frac{\sum_{l=1}^{j}r_{il}I\left(G_{\left(l\right)}\in p_{\left(t\right)}\right)}{\sum_{l=1}^{p}r_{il}I\left(G_{\left(l\right)}\in p_{\left(t\right)}\right)}-\frac{\sum_{l=1}^{j}I\left(G_{\left(l\right)}\notin p_{\left(t\right)}\right)}{q-|p_{\left(t\right)}|}, \end{array}$$ (5)

where *p*_(*t*)_ is the set of genes in pathway *t*, *I*(*G*_(*l*)_ ∈ *p*_(*t*)_) the indicator function that outputs 1 if the *l*-th gene is a member of pathway *t* and 0 otherwise. *q* refers to the number of genes in the dataset. *d*_*ijt*_ in Eq 5 is computed for each sample, gene and pathway. The gene with a high *r*_*il*_ value starts calculation and *d*_*ijt*_ value shows the difference between genes belonging to the pathway and genes not. The smallest *d*_*ijt*_ is subtracted from the largest *d*_*ijt*_, which is the mES of pathway *t* in sample *i* (Eq 6),

$$\begin{array}{c} mES_{it}=\underset{j=1,\ldots ,n}{\text{max}}(0,d_{ijt})-|\underset{j=1,\ldots ,n}{\text{min}(0,d_{ijt})}| \end{array}$$ (6)

Instead of using integer ranks as in GSVA, the continuous values ${\vec{r}}_{i}$ are used to reflect the difference of multi-omics features.

Second, to compute the omics contribution rate OCR, two types of input data are required: 1) the *S*, *G* and *O* components and 2) the sample assigned features $\hat{f}$. The contribution of each omics towards the mES is used to interpret how much the pathway is influenced by which omics. Since OCR aims to reflect the activity of omics, it is advantageous to observe it per clinical feature group so that the differently activated omics across the groups can be compared instead of individual samples. Thus, OCR is computed for each clinical feature group, where features that are commonly assigned to samples within a same group are collected. Features shared by 50% of the samples in a group were collected from *S*′, which are expected to represent the *m* group’s specific multi-omics profiles and denoted as ${\hat{f}}_{m}$. Experimental evaluation results are shown in the results section to show that the assumption of feature sharing tendency is observed in colon and stomach cancer cohorts. The strongest associated feature of each gene is selected from $g_{j}^{{}^{\prime }}$ for every gene in *p*_(*t*)_. The omics profiles of features $O_{{\hat{f}}_{m}}^{\prime }$ are then summed to compute ${\vec{C}}_{mt}$, which is a vector of length *L* (or number of omics types), using Eq 7.

$$\begin{array}{c} {\vec{C}}_{mt}=\sum_{j=1}^{p_{\left(t\right)}}O_{argmax(g_{j{\hat{f}}_{m}})}^{\prime } \end{array}$$ (7)

The OCR for pathway *t* of group *m* for each omics type *k* is obtained by

$$\begin{array}{c} OCR_{kmt}=C_{kmt}/\sum_{k=1}^{L}C_{kmt} \end{array}$$ (8)

mES embeds each gene’s multi-omics profile per pathway and per sample. Thus, mES is computed without any knowledge of hidden groups in the sample dimension, such as subtypes, gender or cluster. A high mES implies that genes related to the selected features are enriched in the pathway of interest. Each selected feature also has an associated multi-omics profile. Collectively, in biological terms, a high mES implies that genes with a specific multi-omics profile are enriched within the pathway of interest. In the other hand, OCR aggregates group specific features to represent the ratio of their multi-omics profiles per group. Thus, the mES and OCR reflect different biological meanings. While mES reflects the enrichment of a certain multi-omics profile of genes within a pathway in a single sample, OCR shows the activation ratio of each omics for the enriched multi-omics profile in a sample group.

#### Step 3: Downstream analysis using mES and OCR for aiding biological interpretation

MOPA provides a number of features to aid interpreting mES and OCR in respect to the biological context of interest. First, survival analysis using mES is supported to further select survival related pathways. For each pathway, the top and bottom 20% samples are labeled and output as high and low mES. Second, using Cytoscape [23], a pathway network is constructed per feature group based on the gene set relation between the pathways (left most figure in Fig 3 Step 3). Here, a node is a pathways in form of a pie chart that visualizes the OCR. The border of a node indicates the survival p-value, whereas the size of the node represent the number of genes in the pathway. A single pathway network is constructed for each sample group. Thus, the pathway networks will have the same topology but will have different forms of pie charts, which is convenient to visualize how the OCR differ between them. Third, to observe if mES represent feature group specific multi-omics characteristics, UMAP (Uniform Manifold Approximation and Projection for Dimension Reduction) [24] and heatmap plots are output. Additionally, a list of ranked genes are output per pathway. Among the genes in a pathway, the significance of their multi-omics profile difference between the clinical feature groups may differ, which can be tested by performing ANOVA analysis on ${\vec{r}}_{j}$, which is the rank weights of gene *j* in all the samples. The *r*_*j*_ is tested whether it is significantly different between the clinical feature group samples (e.g., subtypes). Each gene is tested with Bonferroni corrected p-value. The genes are by the adjusted p-values, which may serve as source for further downstream analysis. The rank of genes implies the multi-omics activity related to the sample *i* associated features, which is expected to be similar across samples of the same clinical feature group.

## Results

### The multi-omics pathway analysis of nine cancer types

To validate the results of MOPA, the association between mES, OCR and clinical features was investigated. Three types of omics were used: gene expression (Ge), methylation level (Me) and miRNA expression (Mi). Here, we tested if the results were coherent with previous biological findings and also made effort to observe what types of clinical features were explainable using mES and OCR.

The results were compared to other pathway enrichment tools, which were GSVA, ssGSEA, z-score and MOGSA. While MOPA is able to utilize clinical label information to compute mES and OCR, the supervised feature selection procedure was skipped for fair comparison since the comparing tools are all unsupervised methods. The unsupervised version of MOPA is referred to as MOPA_UN, which was used for every performance evaluation task involving comparison to other tools. The input data for GSVA, ssGSEA and z-score was the gene expression count matrix of the same samples used by MOPA. For MOGSA, the same data used by MOPA was used as input data, which is the gene-level data.

**Fig 5:**
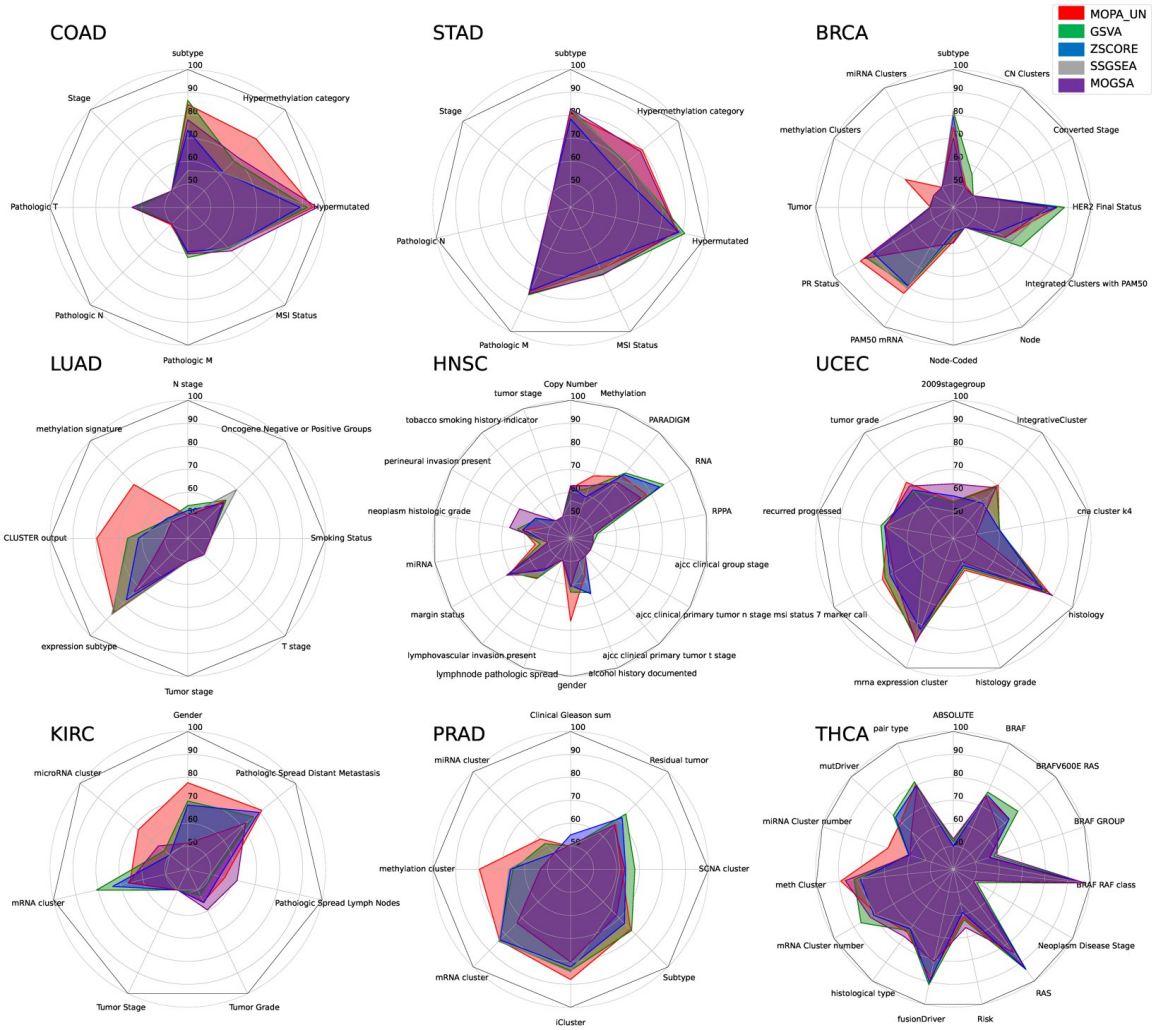
The classification performance comparison of five different pathway scoring methods. The F1 score of 93 different clinical features in nine cancer types was measured for each pathway scoring method.

For each cancer type, MOPA analysis was performed on each clinical feature group. A total of 93 clinical features were tested where MOPA was executed for each feature. By training a Multi-Layer Perceptron (MLP) model on the acquired mES matrix for each clinical feature target label, the 10-cross validated average F1 score of sample classification results was measured. The train and test data were split by 9:1 ratio. The pan-cancer clinical feature group classification results as radar charts in Fig 5. Also, the F1-scores were tested for significance between the methods, which are provided in S2 Table.

From the result, we observed that not all clinical feature groups were well classified. Among the clinical features, subtype, mutation clusters showed high F1 scores in all the tools. MOPA showed the highest F1 scores in COAD, STAD subtypes, mutation and hypermethylation clusters. Single-omics tools also performed well on subtype and hypermutated categories using gene expression data. While MOGSA also utilizes multi-omics data, the performance on the hypermethylation category in COAD was lower than the single-omics tools. In the STAD hypermethylation case, MOGSA performed better than single-omics tools but was below MOPA. A clear advantage of using multi-omics data was observed in classifying the hypermethylation groups in COAD, STAD, LUAD and PRAD, where single-omics tools showed poor performance, which may be due to the difficulty of predicting methylation clusters through the gene expression data. Especially, gender groups were well classified in the HNSC and KIRC datasets by MOPA compared to the other tools. From the results, we learned that noise is accumulated when incorporating more omics types that may hinder learning group specific features. Thus, how to integrate and analyze the heterogeneous multi-omics type is important in terms of not to loose structural information of each individual omics and further learn relations among them. For performance evaluation, we used four different classification methods as shown in Table 3. Random Forest (RF), Support Vector Machine (SVM), K-Nearest Neighbor (KNN) and Multi-layer Perceptron (MLP) classifiers were used to predict the clinical features groups in each cancer type. Overall, MOPA_UN achieved higher F1-scores in all the tasks. While gene expression data was used as input data for the single-omics methods, other single-omics data were also used for performance comparison. The results show that utilizing multi-omics data is advantageous over single-omics data (S1 Fig). There were not enough methods to compare besides MOGSA which outputs a pathway activity score per sample. To compare with a state-of-the-art method, the ActivePathways method was used. ActivePathways is a method that computes a p-value of a pathway for each omics in an individual manner. It takes the p-value of each omics entity as input instead of actual expression data, which has to be provided by the user. The choice of statistical test for computing the p-values are not constrained. Then it combines the p-values to acquire a single p-value, which is then used to measure the significance of a pathway’s activity. Thus, it does not consider the cis-relations between the omics elements and also is not able to provide weight-like pathway score. Since the output of ActivePathways is a list of pathways, we made effort to investigate if the results agreed with the pathways output by MOPA. Using the COAD dataset, ActivePathways reported seven pathways, with significant adj. p-values, as shown in Table 4. Then, the mES scores output by MOPA were tested for significance for the same seven pathways for comparison. Among the seven pathways, six agreed to be significant in both methods. The “Lysosome” pathway had the least significant adj. p-value (0.049) in ActivePathways, whose adj. p-value (0.135) was also least significant in MOPA. Collectively, the output pathways by the two methods were found to agree. ActivePathways also reports the omics that has significant influence to the pathway. However, all pathways showed to be influenced by GE (i.e., gene expression) except the “Focal adhesion” and the “Lysosome” pathways. In comparison, MOPA was able to present the influence of each omics in more detail (pie chart ratios) based on the Omics Contribution Score (OCR) metric (S2 Fig).

**Table 3:** The F1-score for classifying molecular subtypes in COAD and STAD were measured using four different classification methods.

Cancer	Group	Method	RF	SVM	KNN	MLP
COAD	Subtype	MOPA_UN	0.822	**0.87**	**0.837**	**0.865**
		GSVA	0.75	0.854	0.71	0.86
		ZSCORE	0.663	0.766	0.804	0.712
		ssGSEA	**0.853**	0.584	0.746	0.560
		MOGSA	0.732	0.487	0.654	0.789
STAD	Subtype	MOPA_UN	**0.818**	**0.856**	**0.818**	0.814
		GSVA	0.71	0.805	0.659	**0.83**
		ZSCORE	0.673	0.766	0.65	0.791
		ssGSEA	0.78	0.584	0.776	0.83
		MOGSA	0.793	0.466	0.727	0.826
COAD	Hypermethylation cluster	MOPA_UN	**0.723**	**0.808**	**0.72**	**0.814**
		GSVA	0.581	0.618	0.53	0.688
		ZSCORE	0.513	0.659	0.401	0.614
		ssGSEA	0.673	0.555	0.641	0.48
		MOGSA	0.671	0.273	0.494	0.709
STAD	Hypermethylation cluster	MOPA_UN	0.767	**0.82**	**0.816**	**0.808**
		GSVA	0.56	0.68	0.615	0.693
		ZSCORE	0.57	0.66	0.531	0.684
		ssGSEA	0.612	0.543	0.657	0.708
		MOGSA	**0.783**	0.446	0.722	0.778

**Table 4:** Comparison btween ActivePathways and MOPA.

Pathway term	ActivePathways adj. p-value	ActivePathways Supported omics	MOPA adj. p-value
Focal adhesion	3.335E-4	ALL	1.055E-27
ECM-receptor interaction	5.856E-4	Gene	5.341E-25
Axon guidance	8.269E-3	Gene	1.002E-24
Protein digestion and absorption	1.979E-2	Gene	1.522E-30
AGE-RAGE signaling pathway in diabetic complications	2.540E-2	Gene	4.334E-17
Osteoclast differentiation	3.981E-2	Gene	1.716E-38
Lysosome	4.944E-2	Methylation, miRNA	1.348E-1

While the performance of MOPA is not superior, its performance was at least equal or higher than the comparing methods. Furthermore, the mES and OCR metrics are able to interpret the pathways with richer multi-omics context, which will be discussed later.

**Fig 6:**
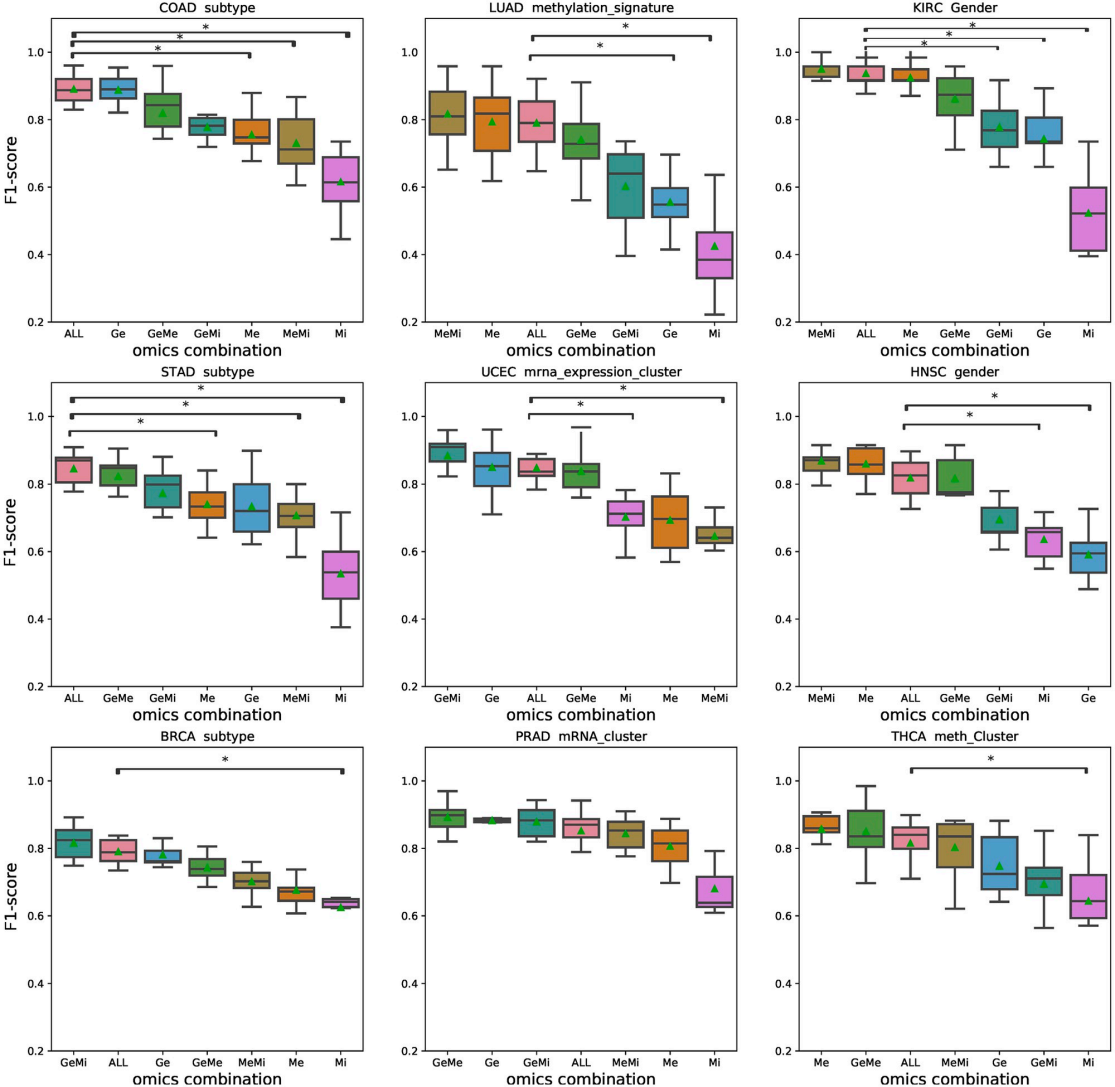
The comparison of omics combination. For each cancer type, the F1 score was measured on the clinical feature with best classification accuracy by utilizing different sets of omics combinations. ‘ALL’ indicates the combination where all three omics were used. Overall, ‘ALL’ showed robust performance.

We further investigated how the classification performance was effected by different combinations of omics. For the same pan-cancer dataset, all possible combinations of omics, including single-omics, were generated and tested, which is provided in Fig 6. As expected, the multi-omics landscape differed between the cancer types and omics combinations, which showed specific combinations to perform better than others. The adj. p-values for each task in provided in the S3 Table. However, in every cancer type, utilizing all omics (i.e., mRNA, miRNA and methylation) showed robust results and the combination of at least two omics showed significantly improved results than single-omics data in all cancer types. For example, the GeMe (i.e., gene expression and methylation omics) combination in STAD showed significant F1 score improvement in the molecular subtype group in Fig 6. Also, here it can be seen that both Ge and Mi single-omics showed lower F1 scores compared to the combined GeMi dataset, indicating that gene and miRNA relations together better represent STAD subtype specific features.

### Experimental verification

To compute mES and OCR, a number of hyperparameters are involved, which were tested for possible fine tuning.

#### Parametric test of mES

mES depends on two hyperparameters. The first is the number of features in the input data. The input to MOPA is the decomposed loading matrices output by MONTI, which requires a user provided *R* value that determines the number of features in the decomposed components. By observing the changing residual of the decomposed matrices using a range of different *R* values, an optimal *R* was chosen based on the elbow method for each cancer type. As shown in S3 Fig, the *R* differed in the COAD, STAD and LUAD cohorts. Interestingly, a large *R* was expected to yield better performance since more features are learned, which was not the case. *R* values of 160 and 200 showed good performance in the three cancer types.

The second is the set of selected features $\hat{f}$, which is a subset of features from the input data and is determined by the CDF threshold. After fixing *R*, a range of CDF thresholds were tested. A higher threshold causes less number of features to be selected, which will cause loss of information when computing mES. In the other hand, a low threshold will select many of features that will weaken clinical feature group specific features. A threshold of 0.6 showed the most robust results in the three cancer cohorts, as shown in the S4 Fig.

#### Evaluation of OCR

**Fig 7:**
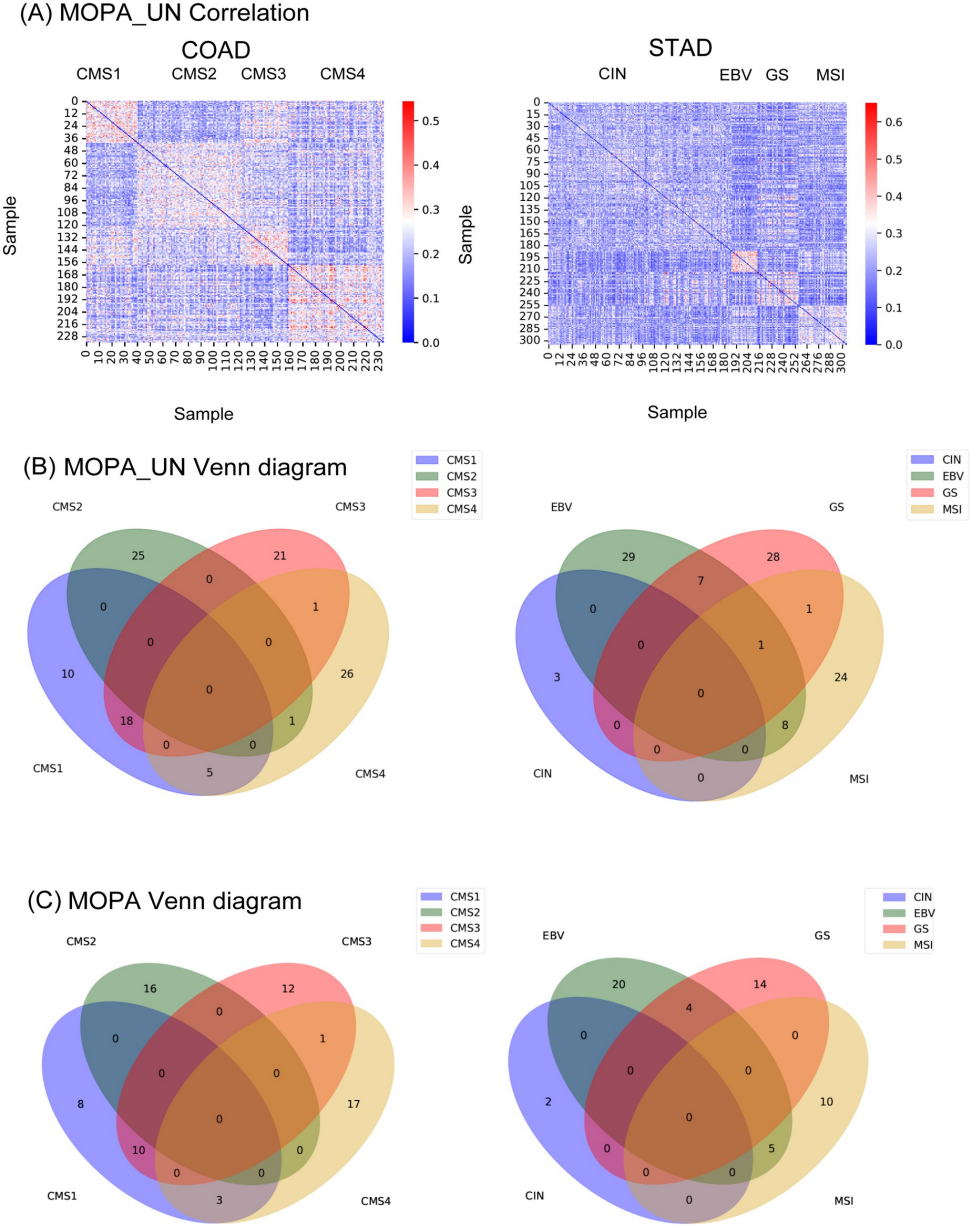
Evaluation results for the unsupervised task. (A) The jaccard similarity of sample features of COAD and STAD subtypes without using labels are shown. (B) The proportion of group shared and group specific features in COAD and STAD in MOPA_UN. (C) The proportion of group shared and group specific features in COAD and STAD in MOPA using label information.

OCR is computed based on the features shared among the samples within the same clinical feature group. Such criteria is based on the assumption that each group share a common multi-omics profile. To test if this is true, instead of using the shared set of group features ${\hat{f}}_{m}$, all the features $\hat{f}$ were used to measure similarity between the samples with jaccard similarity. As shown in Fig 7, even if we did not limit the set of features to group specific features, subtype sample groups in COAD and STAD similar features.

Such pattern was more evident using the group shared features. The Venn diagram in Fig 7C, shows the overlap of features ${\hat{f}}_{m}$ between the groups, where it can be seen that the majority of features were assigned specifically to each group.

### Performance test without clinical feature labels

**Fig 8:**
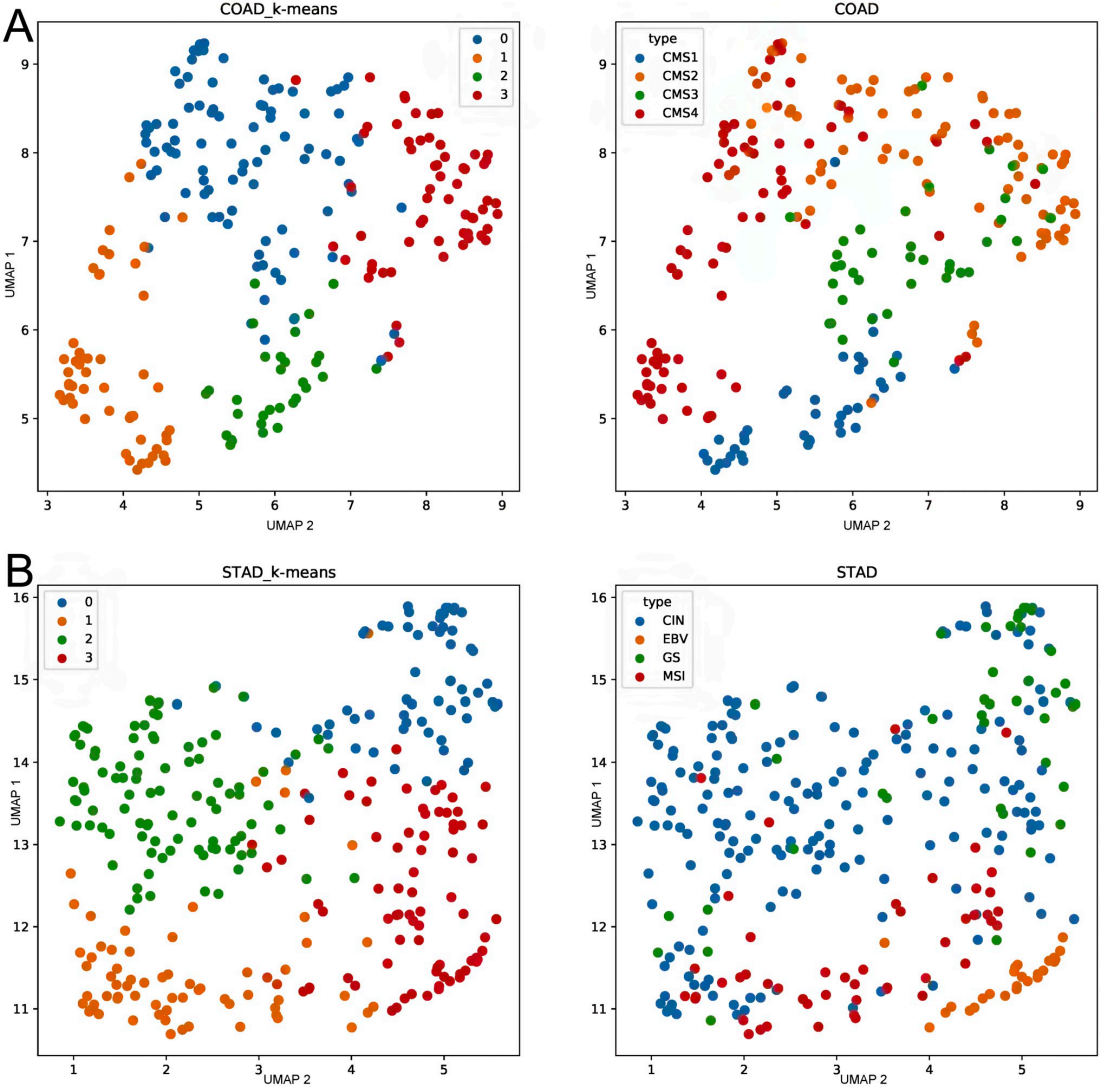
The K-means clustering result using mES of COAD and STAD. The (A) COAD and (B) STAD ground truth subtype labels were well recovered.

**Fig 9:**
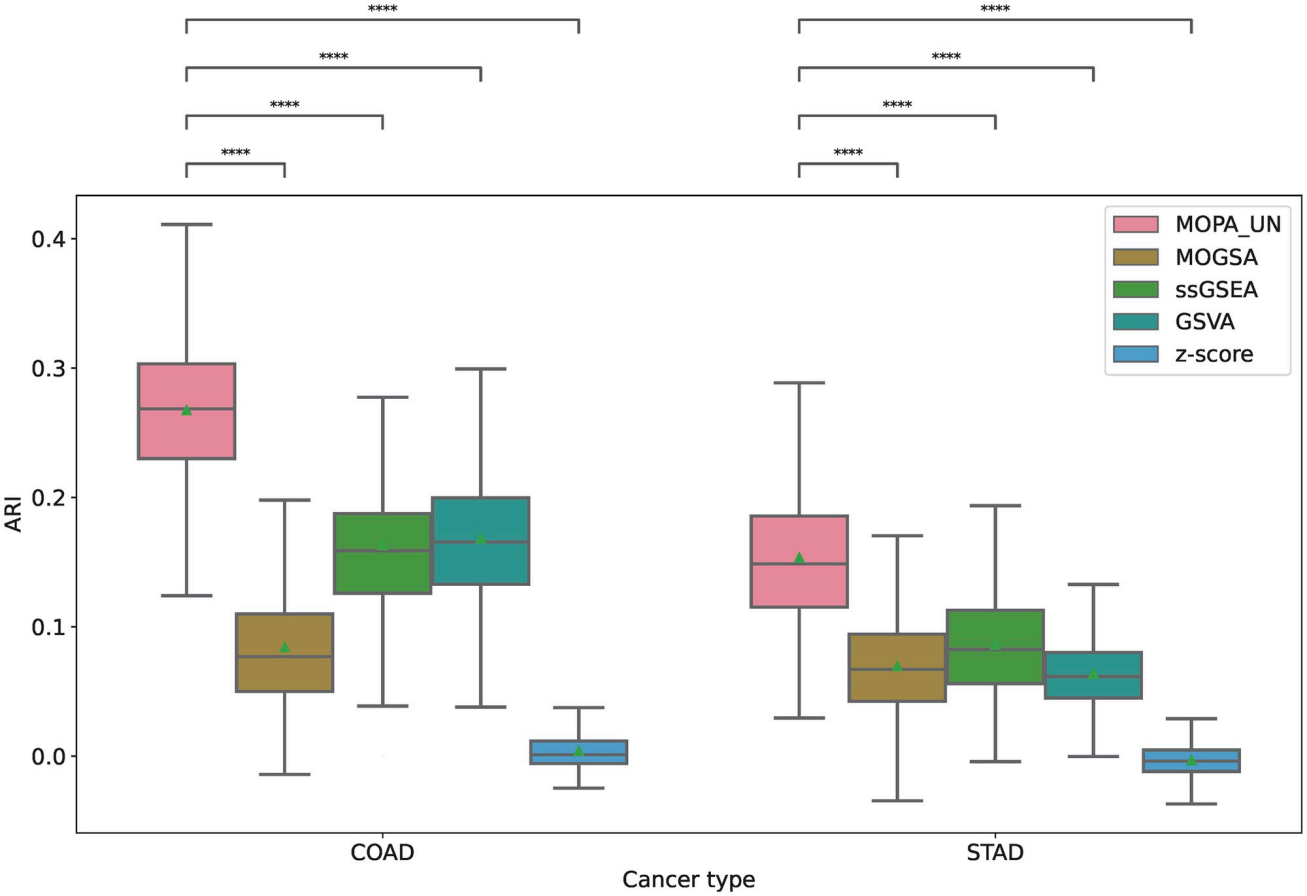
Comparison of the clustering quality. The ARI of COAD and STAD samples clusters was measured using their molecular subtype labels as ground truth labels (color coded).

mES and OCR are computed using the preselected features by MONTI with clinical label information. Such supervised approach limits the analysis to association studies and cannot be used to detect novel sample subgroups. Thus, MOPA was further tested if it was able to detect group specific pathways in unsupervised manner. For such purpose, the feature selection process within MONTI was skipped, so that MOPA only used the CDF threshold for filtering low informative features. The samples were clustered using the mES matrix using the K-means algorithm to detect multi-omics sample clusters. The cluster numbers were then used as labels to measure the classification performance. Similarly, the pathway scores of the comparing tools were used for clustering in Fig 8. The clustering quality was compared by the Adjusted Rand Index (ARI) using the molecular subtypes as ground truth labels since they were shown to be most well discriminated. For robust evaluation, 30% of samples of each subtype group were bootstrap sampled for 1,000 times from which the ARI was measured. As shown in Fig 9, MOPA and the unlabeled version MOPA_UN showed significantly higher ARI scores than the other tools.

**Fig 10:**
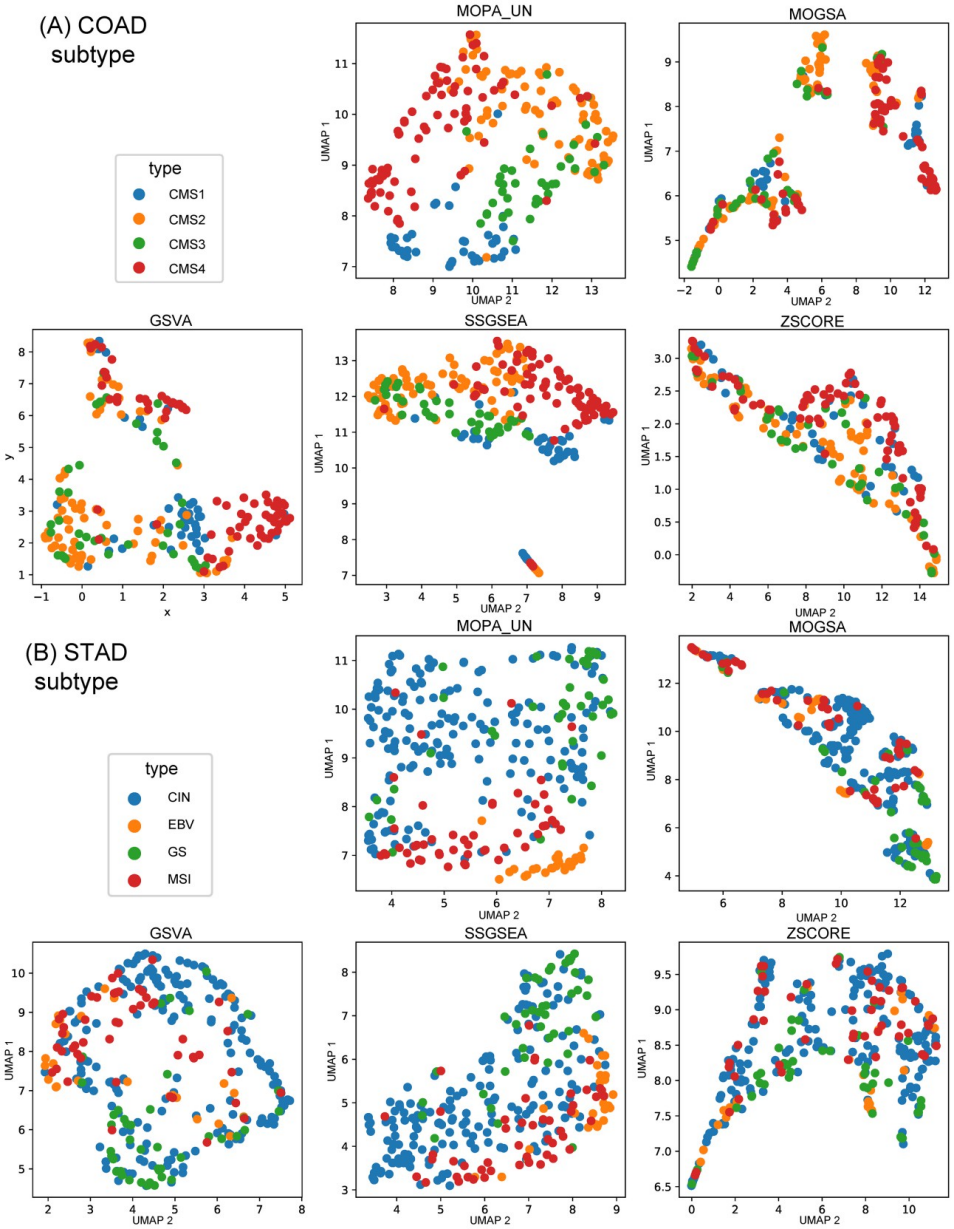
The performance comparison between the five different pathway scoring methods. (A) COAD subtype color coded UMAPs and (B) STAD subtype color coded UMAPs.

Similarly, the UMAP plot indicates that the subtype group samples were successfully identified by MOPA_UN. Meanwhile, ssGSEA also showed a high ARI score and well discrimination of subtype groups in COAD (Fig 10A). Also in STAD, subtype groups were well distinguished (Fig 10B).

In case feature labels are available, MOPA performs feature selection in a semi-supervised manner, which involves the feature selection process of MONTI in addition to the feature filtering by CDF. However, MOPA is able to compute mES and OCR without labels in case clinical feature label information is not available. While mES does not require any label information, OCR does. Thus, by performing simple K-means clustering on the mES matrix, sample clusters can be identified, which will serve as sample group labels that are required to compute OCR. In Fig 10, it can be seen that the actual COAD and STAD sample clusters well recovered the subtype sample groups.

### Use case study

From two case studies, COAD and STAD, we present a detailed analysis procedure based on mES and OCR to show how MOPA was able to reproduce previously reported biologically important results.

#### Use case Study 1—COAD

**Fig 11:**
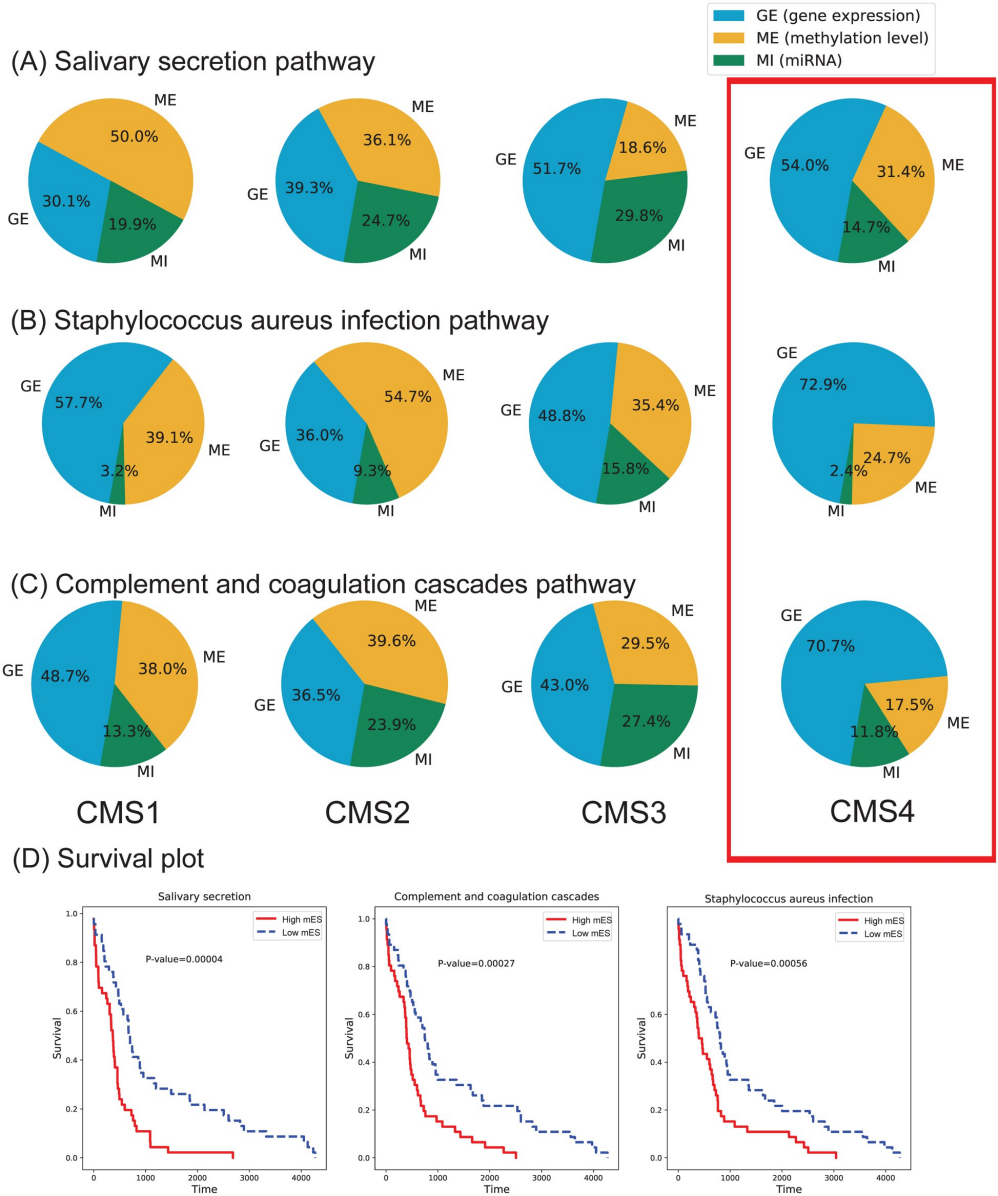
The OCR and survival plots of three pathways are shown for each COAD subtype. (A), (B) and (C) represent the OCR of the pathways, respectively. (D) The survival plots of the three pathways.

From the COAD data, a total of 106 pathways with significant survival p-values were detected to be associated with COAD molecular subtypes. For the survival analysis, two group of samples were defined in terms of the mES score. The top 20% and bottom 20% samples in respect to the mES score were defined as high mES and low mES groups. As shown in Fig 11D, the most significant pathways were the ‘Salivary secretion’, ‘Complement and coagulation cascades’ and ‘Staphylococcus aureus infection’.

To reason why these three pathways were selected, we investigated how the high mES and low mES groups were associated with the COAD CMS1, CMS2, CMS3 and CMS4 molecular subtypes. The number of samples and percentage per subtype were counted in each high and low mES groups as show in Table 5. The sample composition of all pathways are provided in the S4 Table. Here we observed that the subtype sample composition significantly differed between the subtypes. Especially, the majority of CMS4 samples were found in the high mES group, while CMS2 and CMS3 samples were both found in the low mES groups in the three pathways. Whereas, CMS1 did not show clear preference to either group, which implies it is located between the high and low mES range. Furthermore, it is observed that the sample composition of CMS2 and CMS3 subtypes are similar. Such observation was also reported in the previous study [25], where it was shown that the CMS2 and CMS3 subtypes were difficult to disambiguate. While such result only used gene expression data, other studies showed that the two subtypes had distinctively different methylation profiles [26]. Such observation was also detected by MOPA’s OCR metric, which will be discussed later.

**Table 5:** Percentage of COAD samples belonging to high and low mES groups.

Pathway	mES group	CMS1	CMS2	CMS3	CMS4
Salivary secretion	High	6 (15%)	4 (4%)	4 (10%)	32 (42%)
	Low	6 (15%)	19 (23%)	16 (42%)	5 (6%)
Complement and coagulation cascades	High	5 (12%)	0 (0%)	0 (0%)	41 (54%)
	Low	7 (17%)	24 (29%)	15 (39%)	0 (0%)
Staphylococcus aureus infection	High	14 (35%)	0 (0%)	0 (0%)	32 (42%)
	Low	3 (7%)	27 (33%)	16 (42%)	0 (0%)
TGF-beta signaling pathway	High	2 (4%)	4 (4%)	0 (0%)	40 (53%)
	Low	7 (24%)	20 (24%)	18 (47%)	1(1%)

From the result, we observed that the survival probability of high mES group (or CMS4 samples) was significantly lower than other subtype samples, which was also reported in [27]. While examining the relationships among the pathways, we found that the pathways were related to each other through the ‘TGF-beta signaling’ pathway. The over expression of TGF-beta signaling was shown to influence the salivary gland development [28], while similar studies were also present for the other pathways [29, 30]. While the survival p-value of the ‘TGF-beta signaling’ pathway was not as significant as the others, the high and low mES sample composition was very similar to them.

We further examined the ‘Salivary secretion’ pathway to observe what genes contributed to the mES score the most. Each gene of the pathway were subject to ANOVA testing using ${\vec{r}}_{i}$, which reflects the mES score in units of genes. As a result, 32 genes showed to be significantly associated with one or more CMS subtypes, among which BEST2 (Bestrophin 2) was the top gene (Bonferroni p.adj = 1.58E-33). BEST2 is identified as one of the methylation markers for detecting the prognosis of colon cancer [31].

**Fig 12:**
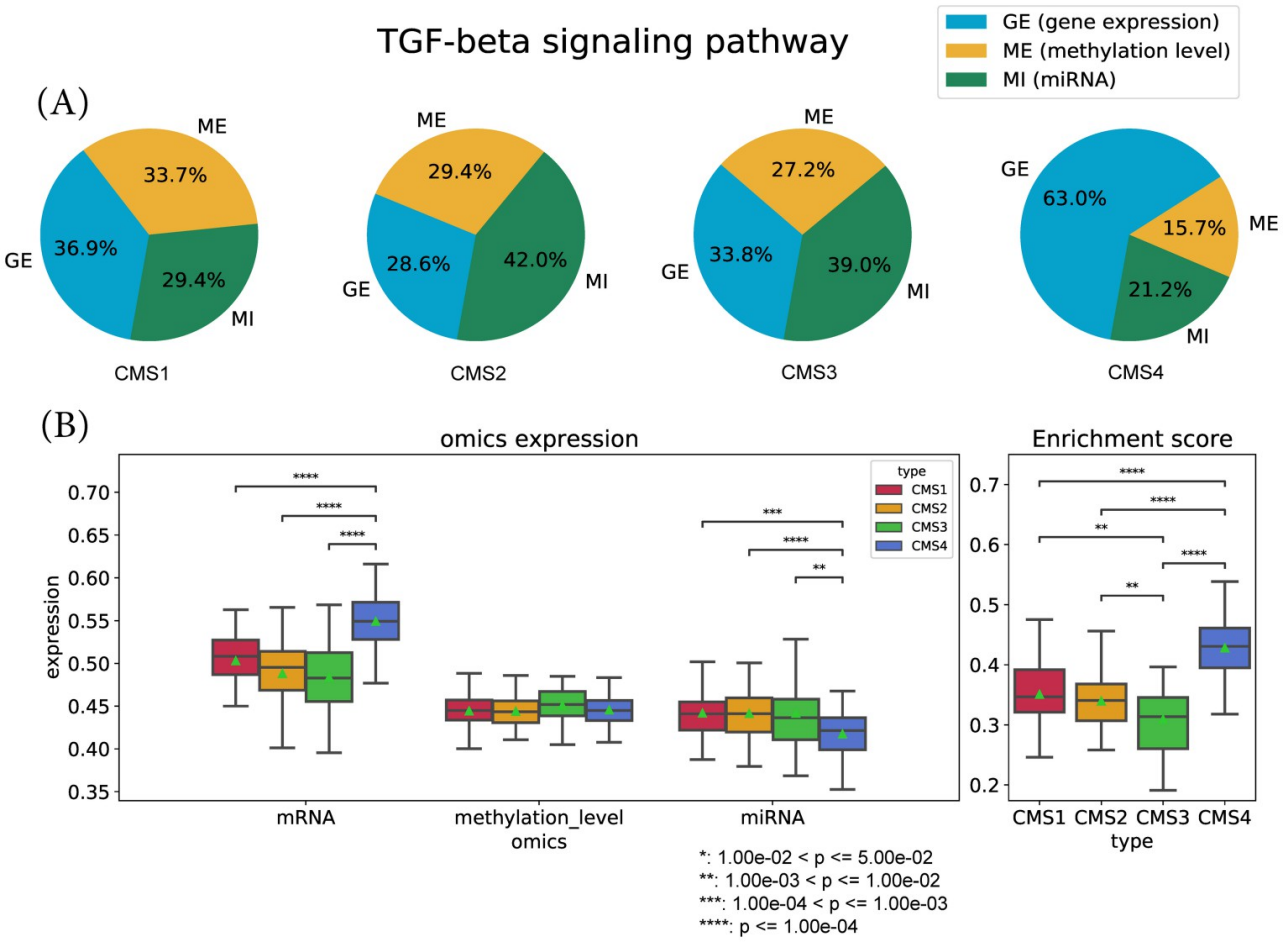
The ‘TGF beta signaling’ pathway analysis results. The OCR and mES of the ‘TGF-beta signaling’ pathway is shown. (A) The OCR of TGF-beta signaling pathway for each subtype. (B) The average gene omics values in the pathway (left) and the mES value for each subtype (right). The statistical significance is indicated.

**Fig 13:**
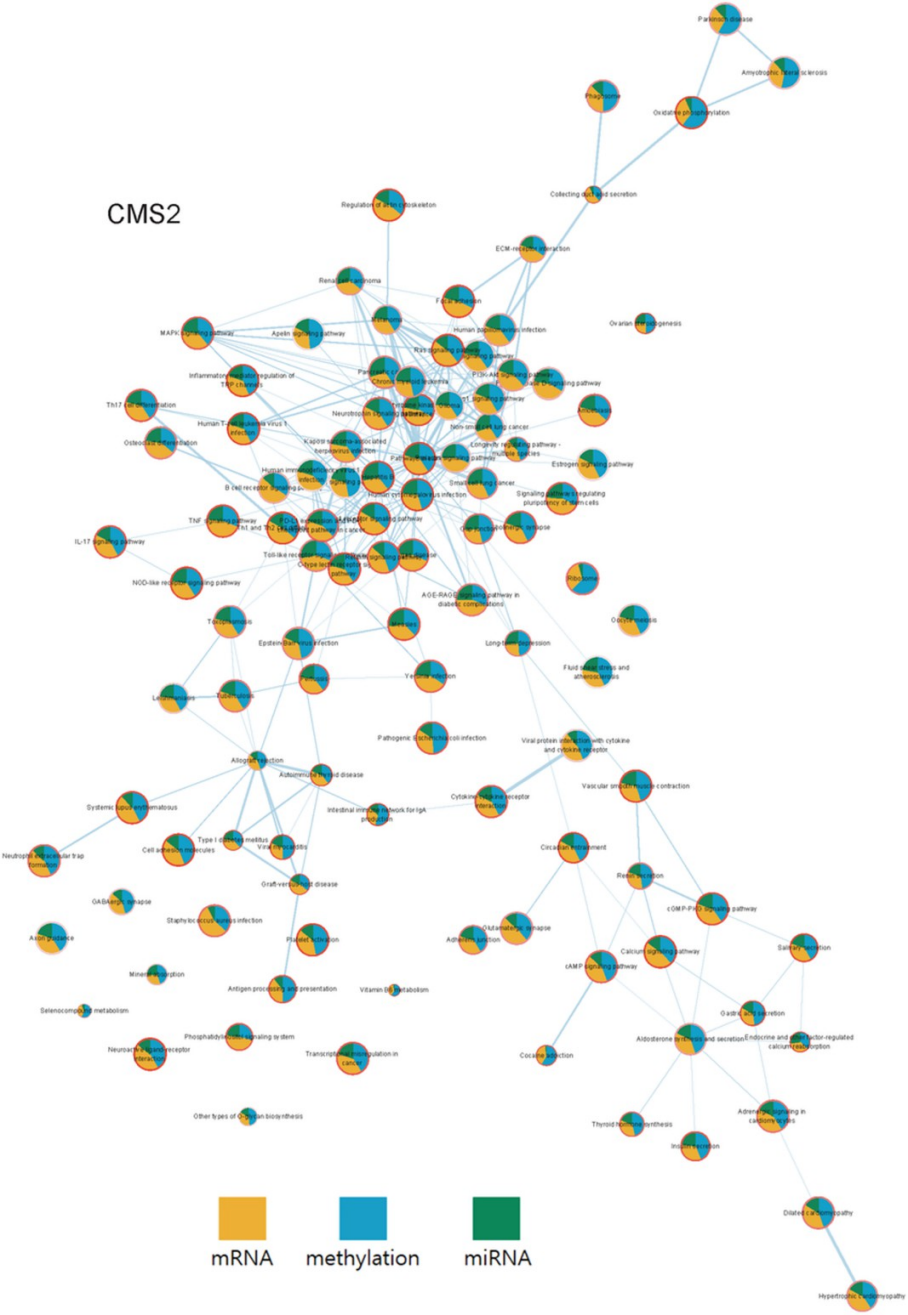
The pathway networks of COAD CMS2. Here, each node represents a pathway, where the pie chart indicates the OCR and the border the survival p-value. Each edge represents how many genes are shared within each pathways (node). The OCR values according to each group were visualized from the COAD data.

According to the OCR of the ‘TGF-beta signaling pathway’ in Fig 12, we observed that the CMS4 subtype had a distinctively different ratio of omics activation. The gene and miRNA expression significantly differed between CMS4 and the other subtypes, which caused CMS4 to have a high mES. This was also observed in three other pathways, which were the ‘Salivary secretion pathway’, ‘Staphylococcus aureus infection pathway’ and the ‘Complement and coagulation cascades pathway’. Collectively, it implies that the CMS4 subtype yields a very different multi-omics landscape in Fig 13.

**Fig 14:**
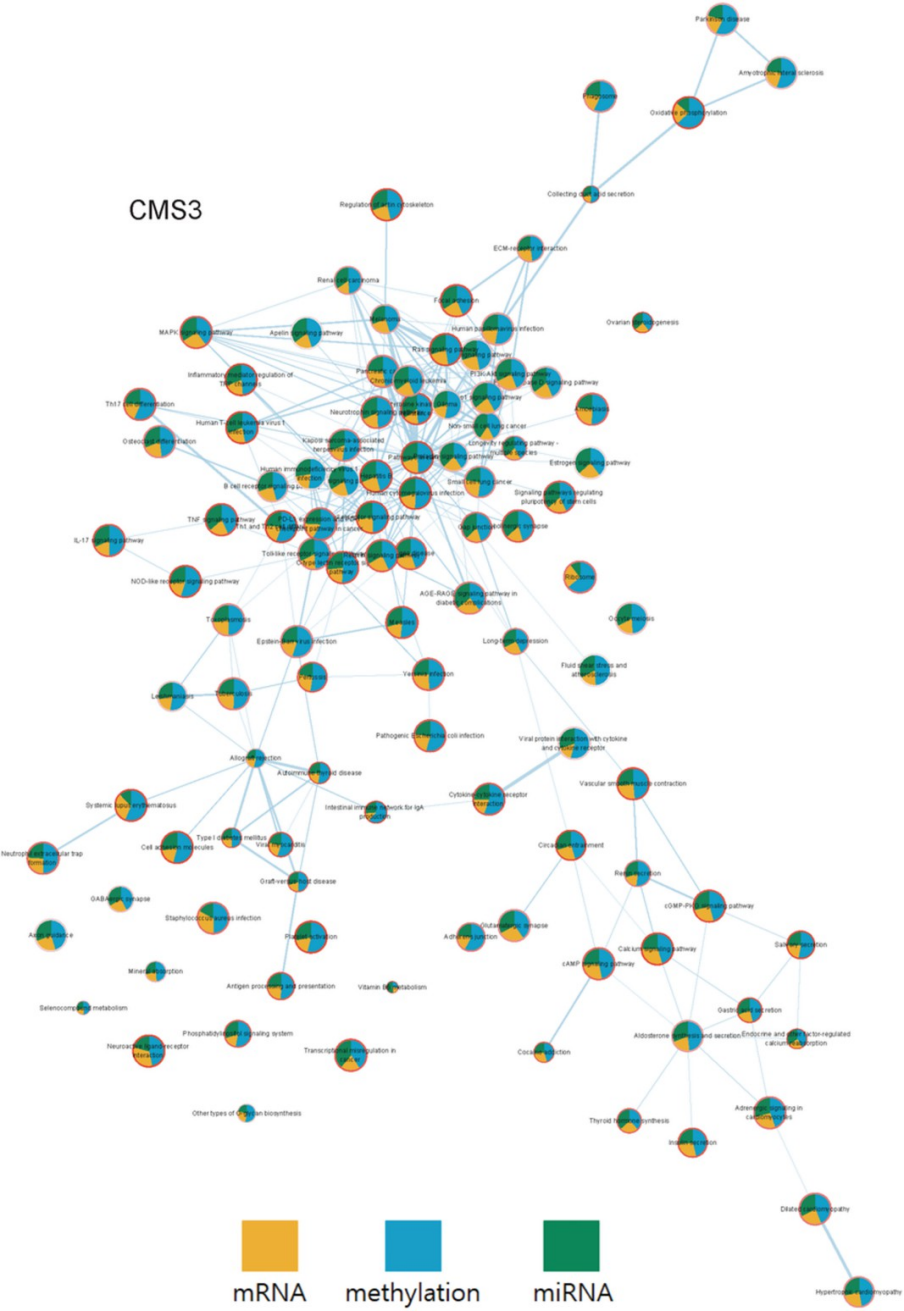
The pathway networks of COAD CMS3. Here, each node represents a pathway, where the pie chart indicates the OCR and the border the survival p-value. Each edge represents how many genes are shared within each pathways(node). The OCR values according to each group were visualized from the COAD data.

**Fig 15:**
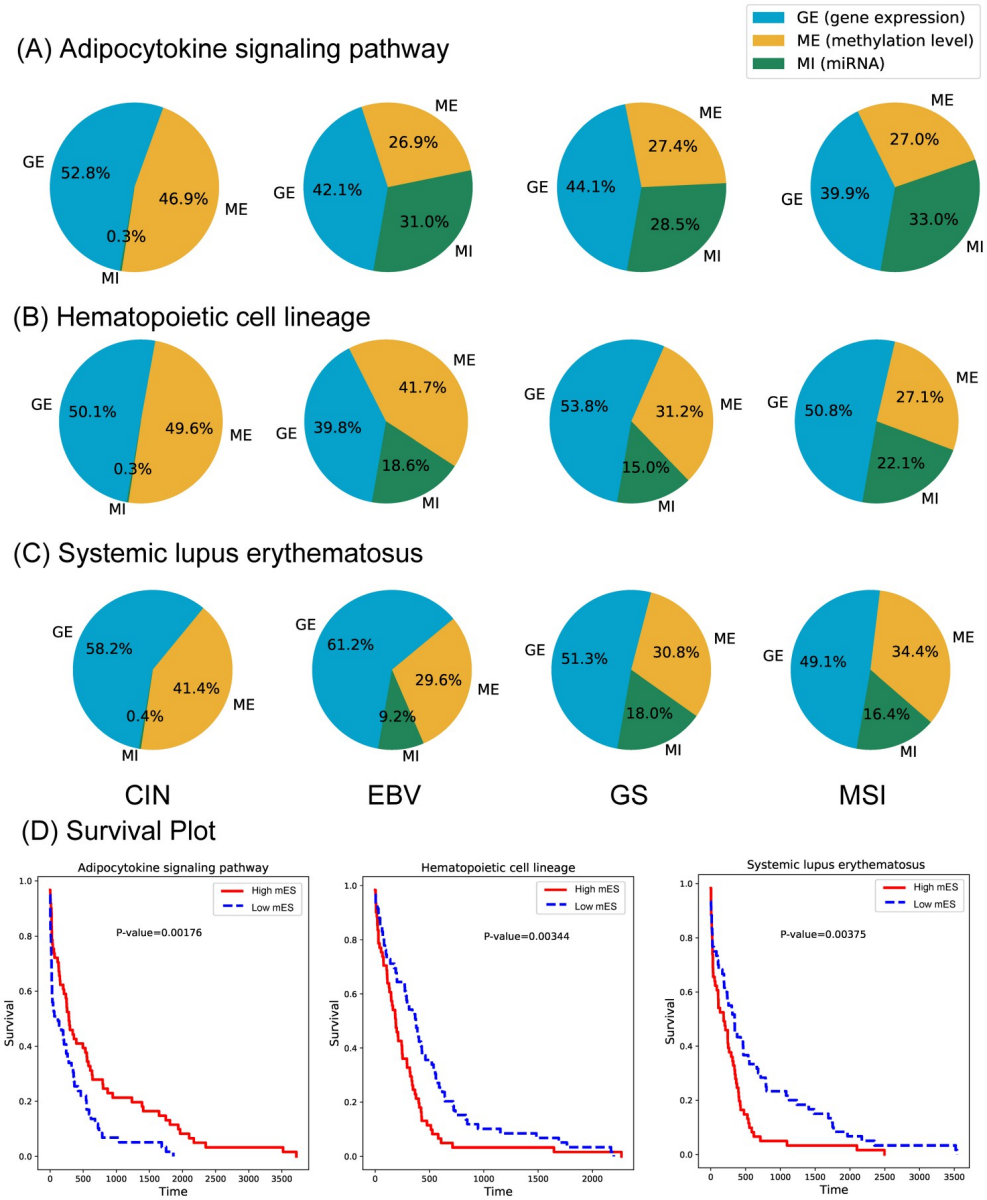
The OCR and survival plots of three pathways are shown for each STAD subtype. (A), (B) and (C) represent the OCR of the pathways, respectively. (D) The survival plots of the three pathways.

To compare the complete set of pathways with significant survival p-values, a pathway network specific to each subtype was constructed using Cytoscape as shown in Figs 14 and 15. From the networks, a clear difference in OCR among the four subtypes was captured. While the gene expression profile of CMS2 and CMS3 did not show significant difference as reported in [26], the two subtypes clearly differed in terms of OCR, especially by methylation.

#### Use case Study 2—STAD

**Fig 16:**
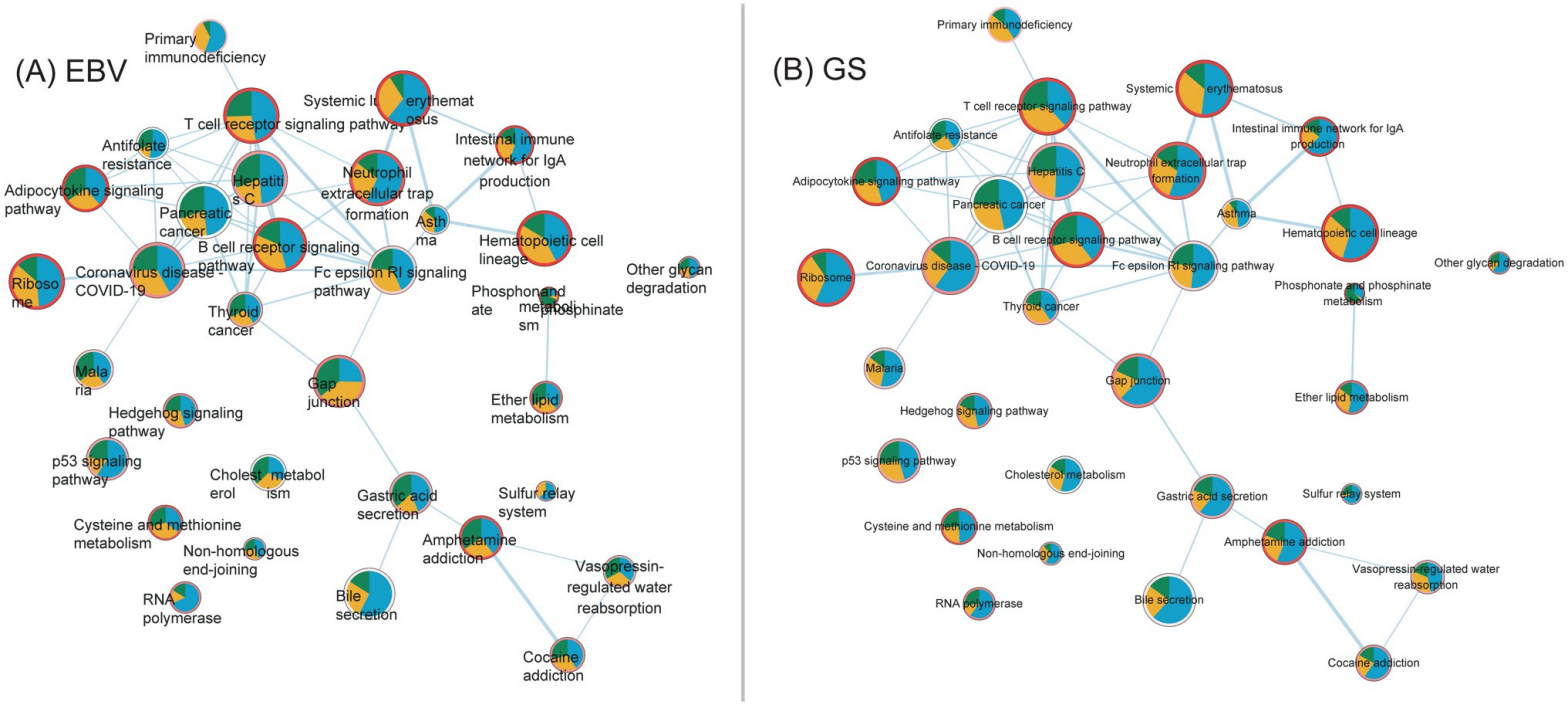
The pathway networks of STAD subtypes. Here, each node represents a pathway, where the pie chart indicates the OCR and the border the survival p-value. The OCR values of the STAD subtypes (A) CIN and (B) GS were visualized.

In the STAD molecular subtype (i.e., CIN, EBV, GS and MSI) data, 34 pathways showed significant survival p-values (Fig 15D). Similar to case study 1, two of the top three pathways showed significant inverse high and low mES sample compositions between the EBV and GS subtypes (S5 Table), which were the ‘Adipocytokine signaling’ and the ‘Systemic lupus erythematosuslineage’ pathways, which are known to be biologically related [32, 33]. The OCR of each subtype of the three pathways are shown in Fig 15. The EBV and GS subtypes were previously reported to have different survival rates [34] Interestingly, the OCR ratio of miRNA in all pathways, including the three mentioned above, was very small only in the CIN subtype. Such observation was also made in the previous [35]. As important genes in the ‘Adipocytokine signaling’ pathway, NFKB1 and STATS were found, which are known to affect the immunity of gastric cancer [36]. Since, the EBV and GS subtypes showed to be very different form each other, pathway networks of the two were constructed and visualized as shown in Fig 16.

Collectively, through the two case studies, we showed that the mES and OCR metrics were useful to interpret the biological functions of the pathways in relation to survival. Both mES and OCR output a result in units of pathways. However, mES takes into account the feature values of each gene with membership to a pathway. Thus, the mES score reflects the multi-omics activity of a certain set of genes. In the other hand, OCR reflects the multi-omics activity in a more broader aspect. If the each different omics are similarly activated in a pathway, the OCR will show equal ratio of each. However, this does not imply that the same genes in the pathway have similar multi-omics activity. Instead, OCR represents the global activity of each omics in a pathway from which we can quickly see what types of omics are effecting the pathway.

## Discussion

MOPA aims to provide a view that aids in interpreting a pathway in terms of omics activation. By comparing two or more conditions, it is difficult to determine how the multi-omics activity differ between them. To provide such insight, the mES and OCR metrics were developed, which reflects the activity of each omics in unit of pathway. Such approach is easier to understand compared to an output that is a list of genes, which requires further enrichment analysis. Using a nine different cancer multi-omics data sets MOPA showed equal or higher performance to competing tools. Through the two case studies, the mES and OCR metrics showed to well reflect previous biological findings of colon and gastric cancer types.

While only three omics types was used in this study, MOPA does not limit the type of and also is not tailored to cancer studies. However, due to the nature of the tensor decomposition technique, MOPA may not perform well in cases where the dataset is small since number of ranks is constrained to the minimum dimension. In case of a small sample dataset, only a few features will be available to work with, which may not be enough to embed the complex multi-omics relations. By rigorous testing with different number of ranks in S5 Fig, we found that at least a rank of 120 was required to capture biological meaningful relations across the three omics types used in this study.

As a drawback, we found that the running time of MOPA was relatively longer than the compared methods. MOPA took approximately 13 minutes when using the COAD data, which was comprised of 14,464 genes, three omics types and 234 patients. ActivePathways came second with approximately nine minutes. The other tools completed under a minute. The longer execution time in MOPA was mainly due to the tensor decomposition task and the mES score computation.

The multi-omics domain is quickly expanding to other domains such as single-cell COVID studies, we expect that the concept of MOPA can also be applied to such large and more complicated datasets. We find that an easy interpretation of high dimensional and complex omics data will be helpful for interpreting the underlying biology in a multi-perspective view.

## Supporting information

S1 Table Pan-cancer dataset. The datasets used in this study.
(XLSX)

S2 Table Adjusted p-values computed for the F1-score difference between methods. (XLSX)

S3 Table Adjusted p-values computed for the F1-score difference between the multi-omics combinations(Ge,Me,Mi). (XLSX)

S4 Table Survival data of COAD. According to mES, the top 20 percent and the bottom 20 percent of samples were chosen and labels as mES and and mES low respectively. They were used for the survival analysis in COAD. The p-value is calculated according to each pathway, and the lower the value, the greater the difference in people’s survival according to mES. It shows how many samples belong to the top or bottom according to each clinical data type.
(XLSX)

S5 Table Survival data of STAD. According to mES, the top 20 percent and the bottom 20 percent of samples were chosen and labels as mES and and mES low respectively. They were used for the survival analysis in STAD. The p-value is calculated according to each pathway, and the lower the value, the greater the difference in people’s survival according to mES. It shows how many samples belong to the top or bottom according to each clinical data type.
(XLSX)

S1 Fig Comparison of F1-score between the multi-omics and single-omics data. (A) compares multi-omics to methylation, and (B) compares multi-omics to miRNA single-omics data. (C) compares multi-omics to methylation, and (D) compares multi-omics to miRNA single-omics data.
(DOCX)

S2 Fig Focal adhesion pathway OCR. (DOCX)

S3 Fig F1-scores with varying rank numbers during tensor decomposition. Depending on the number of samples and cancer, the number of ranks affects performance. Performance was compared according to the number of ranks.
(DOCX)

S4 Fig F1-score according to the threshold. When CDF threshold uses MOPA, it becomes a cut-off criterion for each sample. Performance is significantly affected when it is determined how many rank features are selected for each sample according to this threshold.
(DOCX)

S5 Fig Performance changes according to the number of ranks. The cancer subtype classification accuracy of COAD, and STAD was measured using features selected by the L1 and L2 method with different ranks in MONTI.
(DOCX)
