# Supplementary material for: MOPA: An integrative multi-omics pathway analysis method for measuring omics activity
Source: PLoS One. 2023 Mar 16;18(3):e0278272. doi: 10.1371/journal.pone.0278272 (PMC10019735; doi:10.1371/journal.pone.0278272)
Supplement: S1 Fig — (A) compares multi-omics to methylation, and (B) compares multi-omics to miRNA single-omics data. (C) compares multi-omics to methylation, and (D) compares multi-omics to miRNA single-omics data. (DOCX) [file pone.0278272.s006.docx]

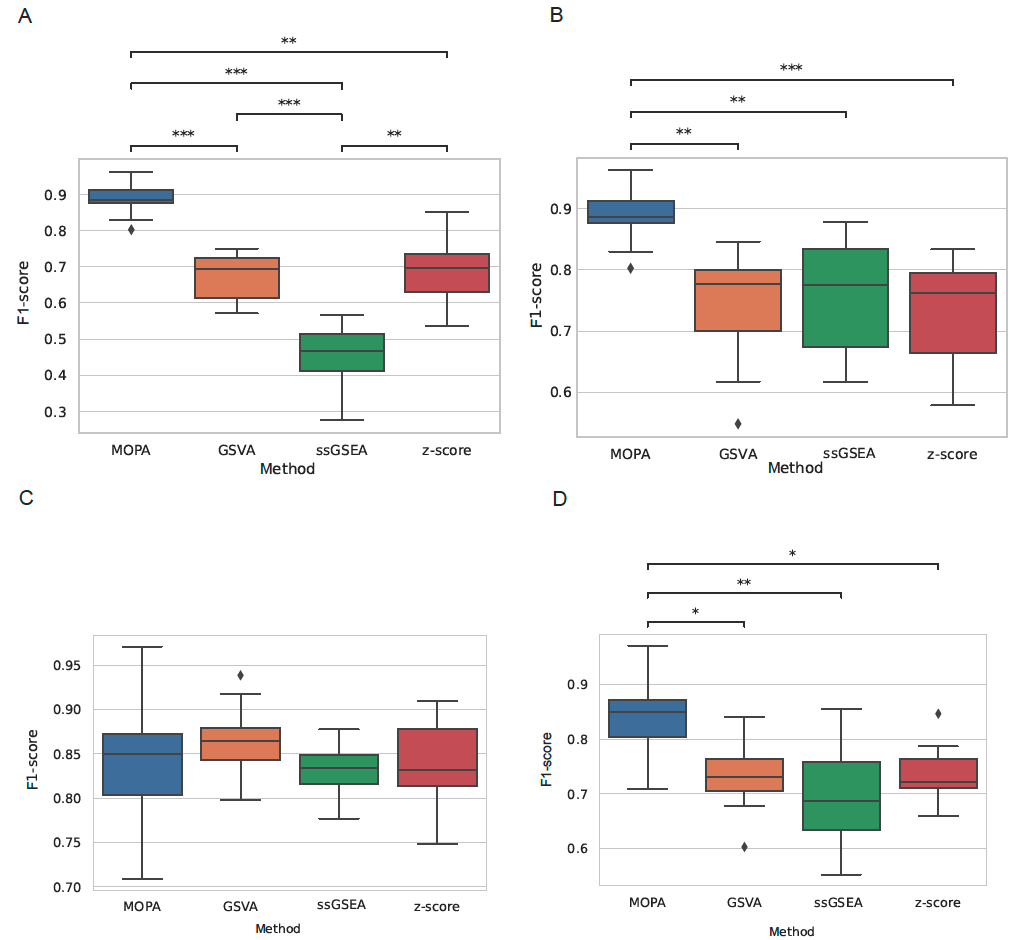


Supplementary Figures S3. Comparison of F1-score between the multi-omics and single-omics data. (A) compares multi-omics to methylation, and (B) compares multi-omics to miRNA single-omics data.  Comparison of F1-score using single omics with STAD data. (C) compares multi-omics to methylation, and (D) compares multi-omics to miRNA single-omics data.
