# Supplementary figures and images for: MOPA: An integrative multi-omics pathway analysis method for measuring omics activity
Source: PLoS One. 2023 Mar 16;18(3):e0278272. doi: 10.1371/journal.pone.0278272 (PMC10019735; doi:10.1371/journal.pone.0278272)

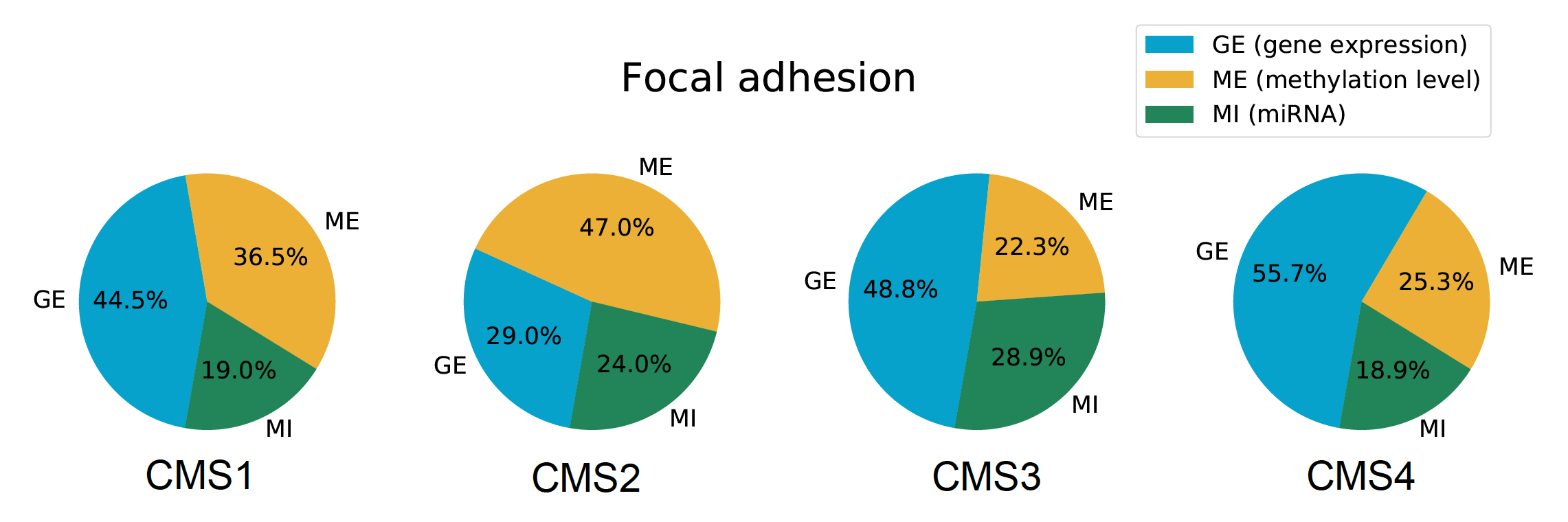


Supplementary Figures S4. OCR of Focal adhesion kegg pathway in COAD subtype.

Supplement: S2 Fig — (DOCX) [file pone.0278272.s007.docx]
