# Supplementary material for: MOPA: An integrative multi-omics pathway analysis method for measuring omics activity
Source: PLoS One. 2023 Mar 16;18(3):e0278272. doi: 10.1371/journal.pone.0278272 (PMC10019735; doi:10.1371/journal.pone.0278272)
Supplement: S3 Fig — Depending on the number of samples and cancer, the number of ranks affects performance. Performance was compared according to the number of ranks. (DOCX) [file pone.0278272.s008.docx]

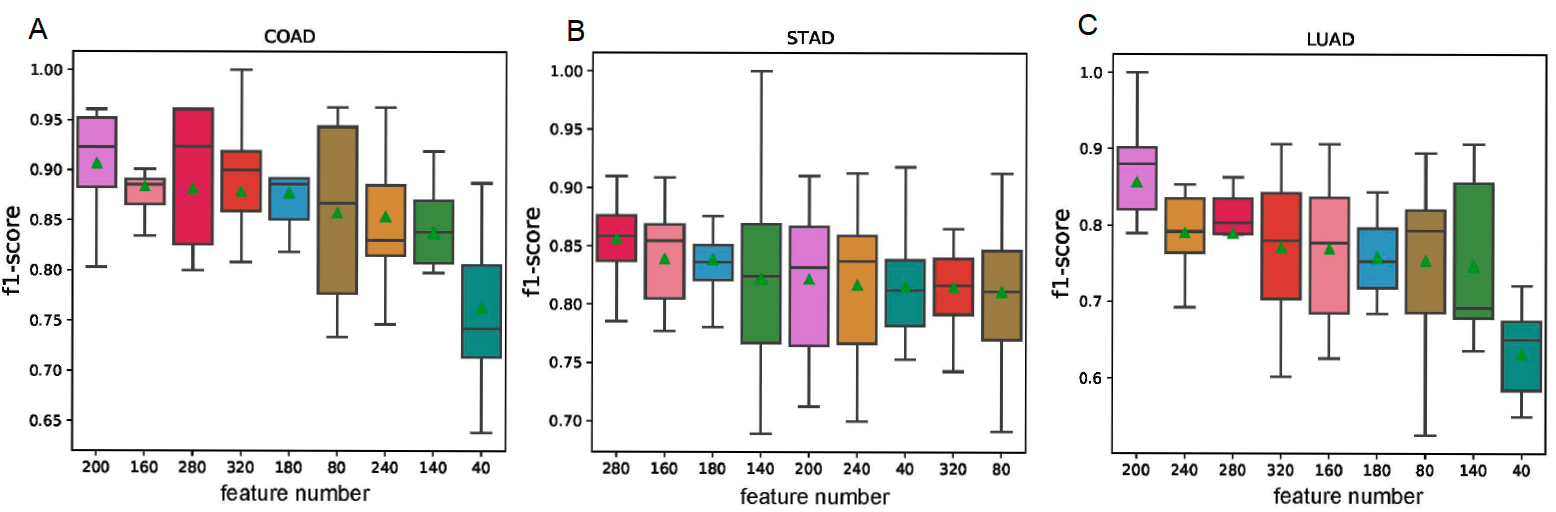


F1-score

Number of ranks

Number of ranks

Number of ranks

F1-score

F1-score

Supplementary Figures S6. F1-score according to the rank number in tensor decomposition. Depending on the number of samples and cancer, the number of ranks affects performance. Performance was compared according to the number of ranks.
