# Supplementary material for: MOPA: An integrative multi-omics pathway analysis method for measuring omics activity
Source: PLoS One. 2023 Mar 16;18(3):e0278272. doi: 10.1371/journal.pone.0278272 (PMC10019735; doi:10.1371/journal.pone.0278272)
Supplement: S4 Fig — When CDF threshold uses MOPA, it becomes a cut-off criterion for each sample. Performance is significantly affected when it is determined how many rank features are selected for each sample according to this threshold. (DOCX) [file pone.0278272.s009.docx]

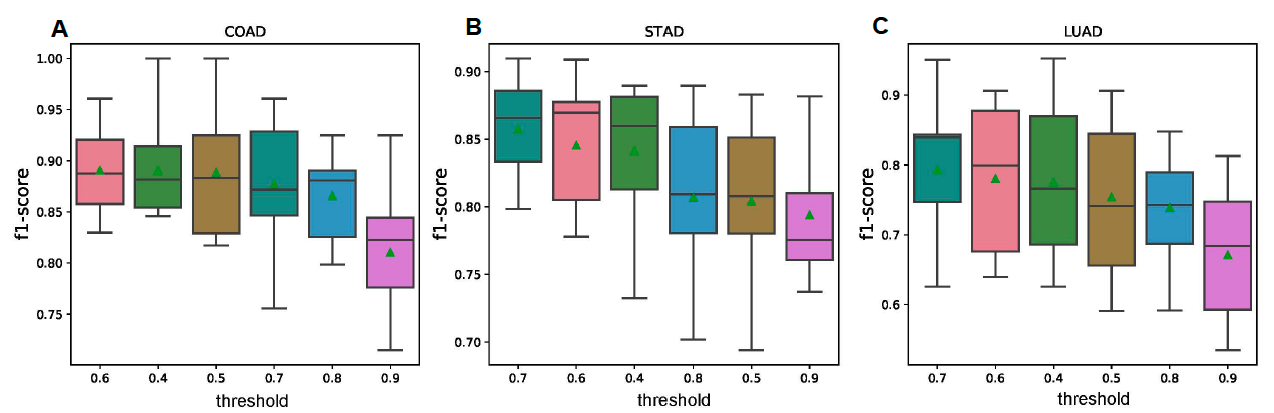


Supplementary Figure S7. When CDF threshold uses MOPA, it becomes a cut-off criterion for each sample. Performance is significantly affected when it is determined how many rank features are selected for each sample according to this threshold.
