# Supplementary material for: MOPA: An integrative multi-omics pathway analysis method for measuring omics activity
Source: PLoS One. 2023 Mar 16;18(3):e0278272. doi: 10.1371/journal.pone.0278272 (PMC10019735; doi:10.1371/journal.pone.0278272)
Supplement: S5 Fig — The cancer subtype classification accuracy of COAD, and STAD was measured using features selected by the L1 and L2 method with different ranks in MONTI. (DOCX) [file pone.0278272.s010.docx]

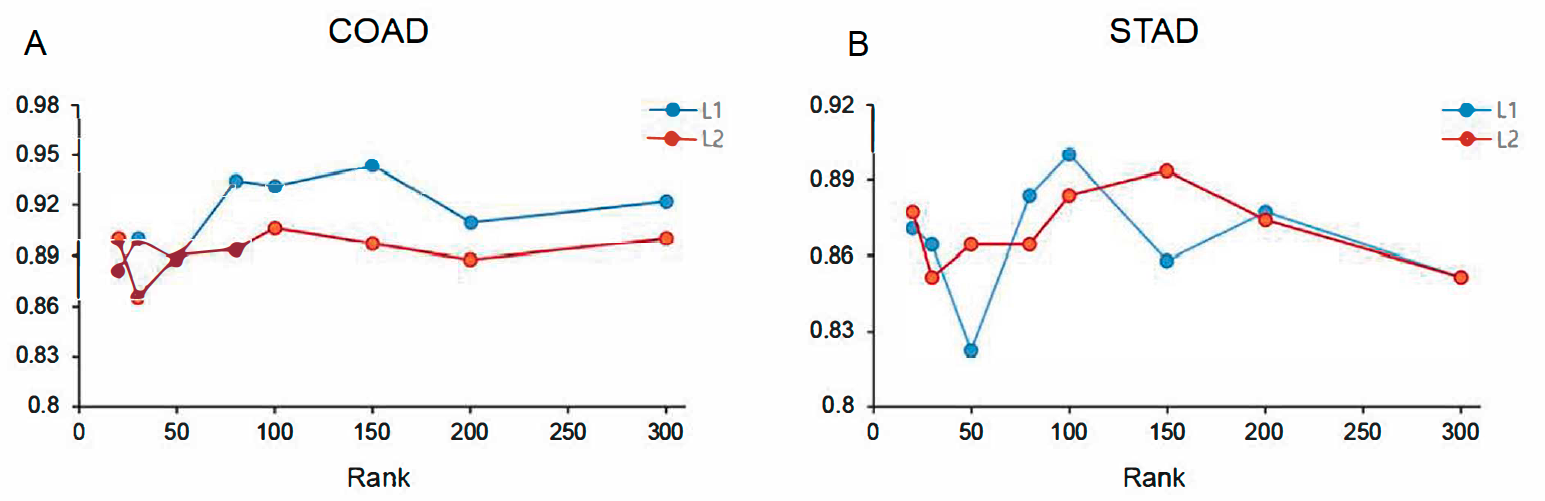


**Supplementary Figure S10.** Performance changes according to the number of ranks. The cancer subtype classification accuracy of COAD, and STAD was measured using features selected by the L1 and L2 method with different ranks in MONTI.
